# Inequality Measurement for Bounded Variables

**DOI:** 10.1002/hec.4969

**Published:** 2025-04-19

**Authors:** Inaki Permanyer, Suman Seth, Gaston Yalonetzky

**Affiliations:** ^1^ ICREA and Centre d’Estudis Demografics (CED‐CERCA) Bellaterra Spain; ^2^ Economics Department Leeds University Business School University of Leeds Leeds UK; ^3^ Oxford Poverty & Human Development Initiative (OPHI) University of Oxford Oxford UK; ^4^ International Inequalities Institute LSE London UK

**Keywords:** bounded variables, consistency, inequality measurement

## Abstract

Many health indicators are bounded, that is, their values lie between a lower and an upper bound. Inequality measurement with bounded variables faces two normative challenges well‐known in the health inequality literature. One is that inequality rankings may or may not be consistent across admissible attainment and shortfall representations of the variable. The other is that the set of maximum‐inequality distributions for bounded variables is different from the respective set for variables with no upper bound. Therefore, the ethical criteria for ranking maximum‐inequality distributions with unbounded variables may not be appropriate for bounded variables. In a novel proposal, we justify an axiom requiring maximum‐inequality distributions of bounded variables to be ranked equally, irrespective of their means. Then, our axiomatic characterization naturally leads to indices that measure inequality as an increasing function of the observed proportion of maximum attainable inequality for a given mean. Additionally, our inequality indices rank distributions consistently when switching between attainment and shortfall representations. In our empirical illustration with three health indicators, a starkly different picture of cross‐country inter‐temporal inequality emerges when traditional inequality indices give way to our proposed normalized inequality indices.

## 1 Introduction

In his seminal contribution, Atkinson (1970) set the foundations of inequality measurement as we know it. After 5 decades, the contributions to this burgeoning field of research have expanded in multiple directions, and “inequality” can arguably be considered one of the most hotly debated topics in an increasingly globalized world, as witnessed by the popularity of several recent books on the subject (e.g., Piketty 2015; Bourguignon 2017; Atkinson 2018; Milanovic 2018; Piketty 2022; Milanovic 2023) and recent awards of the Nobel Memorial Prize in Economic Sciences. The interest in inequality measurement has gone well beyond the study of monetary or pecuniary variables. Like many non‐pecuniary variables, health indicators can only take values from a closed finite interval with fixed limits (i.e., the lower bound and the upper bound).^1^ Following the literature on inequality measurement, we refer to the variables with a lower bound and an upper bound as *bounded variables* (e.g., see Lambert and Zheng 2011).

The measurement of inequality with bounded variables poses two key challenges, which are not relevant for non‐bounded variables (i.e., those with a fixed and finite lower bound but no fixed upper bound). First, when a variable is bounded, one may choose to focus either on the distribution of attainments or the corresponding distribution of shortfalls with respect to the upper bound. For instance, improvements in the coverage of public health programs could be assessed via either the percentage of vaccinated children (an achievement indicator) or the percentage of unvaccinated children (a shortfall indicator). Many well‐known inequality measures fail to rank distributions consistently when measurement switches from attainments to shortfall representations (Micklewright and Stewart 1999; Clarke et al. 2002; Kenny 2004; Erreygers 2009a; Lambert and Zheng 2011; Lasso de la Vega and Aristondo 2012; Bosmans 2016). Several solutions have been proposed in the literature to tackle this challenge, such as using absolute inequality measures (Erreygers 2009a; Lambert and Zheng 2011), indices based on both representations (Lasso de la Vega and Aristondo 2012), or using pairs of weakly consistent indices (Bosmans 2016).

Second, there is a fundamental difference between the conceptualizations of most unequal distribution (henceforth maximum inequality distribution, or MID) for bounded variables and that for non‐bounded variables (i.e., variables with no upper bound), respectively. For a non‐bounded variable, all elements in an MID, barring one, are equal to the lower bound. Inequality measurement in this context is often seen as a cake‐cutting problem (e.g., see Cowell 2011), where the most unequal distribution always involves one person owning the entire cake. For example, while dividing a cake among 10 people, an MID would contain nine people having no slice (e.g., a lower bound of zero) and a single person owning the entire cake. If, instead, there were two identical cakes of the same size, then the MID would feature nine people having no cake at all and one person owning both cakes. As the *mean* increases from one‐10th to one‐fifth of a cake, absolute inequality measures rank the latter MID as more unequal whereas many relative inequality measures rank these two MIDs equally, for instance.

However, such scenario can be infeasible for bounded variables like stunting and immunization rates, all with an upper bound of 1% or 100%. For a bounded variable, an MID becomes a *bipolar* distribution (Erreygers 2009b) or an *almost‐bipolar* distribution (as defined in the subsequent section). To demonstrate our point, we continue with our cake‐cutting example. Suppose there is a fixed upper bound so that no one can have more than half of a cake. When there is one cake, the MID contains eight people having nothing and two people owning half of the cake each (i.e., the upper bound); whereas, with two cakes, the MID features six people without cake and four owning half of a cake each (i.e., leading to bipolar distributions). Thus, while both in the bounded and non‐bounded settings inequality is maximized whenever the smallest share of individuals owns as much as possible, there is a fundamental domain restriction shaping the inequality‐maximizing distributions. In the MID of an unbounded variable a single individual always owns everything, whereas in the MID of a bounded variable such possibility is often precluded. How should we, then, rank the different MIDs in the context of bounded variables?

The health inequality literature has long made inroads into the challenges of assessing inequality with bounded variables (see e.g., Wagstaff 2005; Erreygers 2009a, 2009b; Erreygers and Van Ourti 2011a; Kjellsson and Gerdtham 2013), with proposals for comparing notions of inequality across distributions with different means. For instance, Wagstaff (2005) proposes dividing the concentration index by its maximum value for a given mean; while Erreygers (2009a) proposes absolute indices satisfying various scaling properties, and further proposes normalizing the variable of interest with respect to the corresponding lower‐ and upper bounds to make the interpretations of indices unit‐free. Although the merits of these two approaches and underlying value judgments have been extensively debated (Wagstaff 2011; Erreygers and Van Ourti 2011b), Kjellsson and Gerdtham (2013) seek to reconcile them by arguing that even though both approaches attempt to quantify how far a distribution is from the most unequal state, they differ in their definitions of such states.

However, we observe two gaps in this otherwise rich debate. First, despite independently mentioning some of the elements underpinning our normative proposal for ranking MIDs, the debate has not put forward our ethical justification to rank MIDs equally vis‐à‐vis each other in the context of bounded variables; namely, that inequality cannot increase applying regressive transfers to these distributions. Second, despite similar measurement aims, the debate in the health economics literature has not yet been connected to a related broad (and mostly older) inequality measurement literature (e.g., Temkin 1986; Fields 1987, 1993, 1998; Amiel and Cowell 1994; Bosmans 2007).

Building on alternative suggestions from the literature on non‐bounded variables for similar situations (Temkin 1986; Fields 1998; Bosmans 2007), we argue that, from an egalitarian perspective, the different MIDs in the context of bounded variables represent the normatively *least desirable* situations for correspondingly different means, because in each one of them further regressive transfers are unfeasible. Thence, our normative proposal states that all least desirable situations (i.e., the MIDs) should be considered equally unequal. We refer to this desideratum as the *maximality principle*. Our axiomatic characterization shows that the maximality principle naturally leads to two novel classes of *normalized inequality measures*, which are motivationally analogous to the normalized concentration indices of Wagstaff (2005) and Erreygers (2009b).^2^

Simultaneously, indices in both new classes address traditional concerns regarding the consistency of inequality rankings to alternative representations of the bounded variable (i.e., attainment vs. shortfall) by satisfying the *strong consistency* property (Lambert and Zheng 2011; Bosmans 2016). As an additional contribution to this literature, we show that fulfillment of the more stringent *perfect complementarity* property (Erreygers 2009a) is not just sufficient but also necessary for the fulfillment of strong consistency. Our two classes of indices also comply with *cardinal invariance* (Erreygers 2009a), a well‐established principle in the inequality measurement literature which enables comparisons of distributions with different means for interval and ratio‐scale data, and guarantees consistent inequality rankings in terms of *unit consistency* (Zheng 2007; Lambert and Zheng 2011) and *cardinal consistency* (Erreygers 2009a; Lambert and Zheng 2011).

The key distinction between our two proposed classes is that one is defined for a fixed population, while the other class, a novel contribution to the literature, enables comparisons of distributions with varying populations. The second class' novelty resides in its joint satisfaction of the maximality principle, strong consistency and the population principle (along with other standard properties). Moreover, a key feature setting both classes apart is that the relevant MIDs for fixed populations admit the case of so‐called almost‐bipolar distributions alongside the better‐known bipolar counterparts. By contrast, one of our key contributions is showing that inequality comparisons of bounded‐variable distributions with varying population sizes only admit bipolar distributions as MIDs when they comply with the population principle.

To illustrate the empirical relevance of our proposed normalized inequality measures, we study cross‐country inequality trends in under‐5 and infant survival rates between 1950 and 2015, plus the evolution of inequality in hepatitis‐B immunization rates between 1990 and 2012. For all of them, we compare the normalized standard deviation against its relative (coefficient of variation) and absolute (standard deviation) counterparts. For the two child survival rates the values of the corresponding absolute and relative indices decline throughout, along with increases in mean attainments which were already > 50% in the middle of the 20th Century. By contrast, the proposed normalized index remains stable in value until the end of the 20th Century and falls thereafter. This indicates that observed absolute and relative inequality grew at similar rates compared to their respective maximum possible values during the 20th Century and then at faster rates into the 21st Century. Meanwhile for hepatitis‐B immunization rates, when we use the relative index, inequality steadily decreases throughout, as mean attainment increases from near 0%–90% across the studied period. Instead, we observe Kuznets curves with the absolute index, whereby cross‐country inequality initially increases as mean attainment increases, reaches maximum around half way between both bounds and then decreases as mean attainment approaches its upper bound. However, the normalized standard deviation reports stable inequality values initially and then a fall, suggesting an initial stability followed by a decline in the realized proportion of maximum possible inequality for both absolute and relative inequality.

The rest of the paper proceeds as follows. Section 2 introduces the notation, definitions and the required well‐established properties in the inequality measurement literature. Section 3 discusses the concept of maximum inequality in the context of bounded variables and introduces the maximality principle. Sections 4 and 5 introduce and axiomatically characterize the two classes of normalized inequality measures. Section 6 provides some comparative insights of our proposed approaches in relation to the existing approaches. Finally, Section 7 presents the empirical illustration and Section 8 concludes with some remarks.

## 2 Notation and Well‐Established Principles of Inequality Measurement

Suppose, there are n units of analysis (e.g., people, households, municipalities, countries, etc.) such that n∈N\{1}.^3^ Let $x=\left(x_{1},\text{…},x_{n}\right)$ be an *attainment distribution* of n units (or an n‐dimensional *attainment vector*), where $x_{i}\in \left[L,U\right]\cap Q_{+}$ represents unit i’s cardinally measurable attainment, which is rational and bounded between a non‐negative lower bound of $L\in Q_{+}$ and some positive upper bound $U\in Q_{++}$, that is, 0≤L<U.^4^ To simplify notation, let $D=\left[L,U\right]\cap Q_{+}$ denote the range of values that each $x_{i}$ can take, and let $D=\cup_{L\geq 0}\cup_{U>L}\left[L,U\right]\cap Q_{+}$ be the set of all admissible domains (with L and U being rational numbers).

We denote the set of all attainment distributions of size n taking values within D by $X_{n;D}$, the set of all attainment distributions of size n by $X_{n;•}≔\cup_{D\in D}X_{n;D}$, the set of all attainment distributions taking values within D by $X_{•;D}≔\cup_{n>1}X_{n;D}$ and the set of all possible attainment distributions by $X≔\cup_{n>1}\cup_{D\in D}X_{n;D}$. By definition, we exclude egalitarian distributions whose values are all equal to either bound (i.e., (L,…,L) or (U,…,U)) from our domain. The arithmetic mean function evaluated at any x∈X is denoted by μ(x). Furthermore, for any $x\in X_{n;D}$, let $X_{n;D}^{\mu \left(x\right)}$ be the set of all attainment distributions of size n taking values within D and *with the same mean as* x, and for any x∈X, let $X^{\mu \left(x\right)}$ be the set of all possible attainment distributions *with the same mean as* x. Henceforth, we focus on distributions with mean different from either bound, that is, L<μ(x)<U.^5^

Bounded variables can be represented as attainments $x_{i}$ for every unit of analysis i or, alternatively, as shortfalls $x_{i}^{S}=U+L-x_{i}$, which also range between L and U. For example, if $x_{i}$ is the share of healthy individuals in country i, then $x_{i}^{S}$ is the respective share of unhealthy individuals. In this example, L=0 and U=1, so $x_{i}^{S}=1-x_{i}$. We denote the *shortfall distribution* associated with $x\in X_{n;D}$ as $x^{S}=\left(x_{1}^{S},\text{…},x_{n}^{S}\right)\in X_{n;D}$.

We now present the properties that are relevant for our characterization. An *inequality index* $I:X\to R_{+}$ is a *continuous* real‐valued function expected to satisfy two basic properties (Chakravarty 2009): *anonymity* and *transfer principle*, in addition to other well‐established properties. *Anonymity* requires that an inequality index should not depend on a reordering of attainments across units; whereas the *transfer principle* requires that a transfer from a richer to a poorer unit, without altering their relative positions, should decrease inequality (*progressive transfer*); whereas, alternatively, a transfer from a poorer to a richer unit should increase inequality (*regressive transfer*).^6^

***Anonymity:*** For some $x,y\in X_{n;D}$, I(y)=I(x) whenever y=xP for permutation matrix P.^7^

***Transfer principle:*** For some $x,y\in X_{n;D}$, I(y)<I(x) whenever y is obtained from x by a progressive transfer and I(y)>I(x) whenever y is obtained from x by a regressive transfer.^8^

Before discussing more properties, we introduce a type of function which underpins our characterization results. We refer to a real valued function $f:X\to R_{+}$ as *strictly Schur‐convex* if, for some $x,y\in X_{n;D}$, f(y)≤f(x) whenever y is *majorised* by x and f(y)<f(x) whenever y is majorised by x but y is not a permutation of x (Marshall and Olkin 1979, 54).^9^ Moreover, y is majorised by x if and only if the former distribution can be obtained from the latter through a finite sequence of progressive transfers (Arnold 1987). Therefore, strictly Schur‐convex functions play a prominent role in inequality measurement because they satisfy the transfers principle.^10^

The third property, *equality principle*, ensures that inequality is minimal and is equal to zero whenever all units feature exactly the same value, that is, $x_{1}=x_{2}=\text{⋯}=x_{n}$.

***Equality principle:*** For any $x\in X_{n;D}$ and for some $\lambda \in Q_{++}$, I(x)=0 whenever $x=\lambda 1_{n}$.^11^

The fourth property, *cardinal invariance* (Erreygers 2009a), serves two purposes. First, it allows comparing two distributions when one is obtained from the other by changing the unit of measurement. For example, among health variables, mortality rates may be reported either on a 0–1 scale or in percentage terms 0%–100% (i.e., ratio‐scale), whereas body temperature may be reported either in degree Celsius or Fahrenheit (interval‐scale). The property requires that inequality evaluation remains unchanged when a distribution is obtained from another through a positive linear transformation. Second, although this property is sufficient but unnecessary to guarantee the consistency of inequality comparisons to positive linear transformations of the measurement unit, it crucially enables inequality comparisons of distributions with different means.

***Cardinal invariance:*** For any $x\in X_{n;D}$ and for some $\lambda \in Q_{++}$ and $\delta \in Q_{+}$, $I\left(\lambda x+\delta 1_{n}\right)=I\left(x\right)$, where $\lambda x_{i}+\delta \in \left[\lambda L+\delta ,\lambda U+\delta \right]$ for all i.

We note from the definition of cardinal invariance that its satisfaction implies compliance with scale invariance, that is, I(λx)=I(x), whenever δ=0. Likewise, cardinal invariance boils down to translation invariance, that is, $I\left(x+\delta 1_{n}\right)=I\left(x\right)$, whenever λ=1 and δ≠0. Hence, satisfaction of cardinal invariance encompasses arguably the two most popular approaches to inequality measurement with ratio‐scale data: absolute and relative.^12^ An inequality index is *absolute* if its value remains unchanged when the same amount is added to all attainments (i.e., translation invariance); whereas, an inequality index is *relative* if its value remains unchanged when all attainments are altered in the same proportion (scale invariance). Note that the bounds are allowed to change in both cases from $\left[L,U\right]$ to $\left[\lambda L+\delta ,\lambda U+\delta \right]$.

A form of inconsistency arises when inequality orderings of attainment distributions differ from their shortfall counterparts. Different properties have been proposed in the literature regarding the extent to which inequality indices, as well as partial orderings, should consistently rank attainment and shortfall distributions.^13^ The *perfect complementarity* property requires that the value of the inequality index remains unaltered when we switch between attainment and shortfall representations of the same distribution (Erreygers 2009a).^14^

***Perfect complementarity:*** For any $x\in X_{n;D}$, $I\left(x\right)=I\left(x^{S}\right)$.

Likewise, the less stringent *strong consistency* property requires that the inequality ranking should be robust to alternative representations of the variable (Lambert and Zheng 2011).

***Strong consistency:*** For any $x,y\in X_{n;D}$, $I\left(x\right)\leq I\left(y\right)\Leftrightarrow I\left(x^{S}\right)\leq I\left(y^{S}\right)$.

The task of rendering our proposed inequality measures in compliance with strong consistency is facilitated by the remarkable equivalence between strong consistency and perfect complementarity. We know that the latter implies the former. But Proposition 1 shows that strong consistency also implies perfect complementarity.^15^

Proposition 1 An inequality index satisfies strong consistency if and only if it satisfies perfect complementarity.

See Appendix A1.

The aforementioned properties compare distributions with the same number of elements or population sizes. The final property, the *population principle*, enables the comparison of distributions with different population sizes.

***Population principle:*** For some $x,y\in X_{•;D}$, I(y)=I(x) whenever y is obtained from x by a *population replication*.^16^

## 3 Maximum Inequality Distributions (MIDs) and the Maximality Principle

In the previous section, we discussed some well‐established properties for inequality measurement. Here, we introduce a new principle for inequality measurement in the context of bounded variables, which embodies a normative criterion for the ranking of MIDs with different means. Even though the concept of MIDs has been discussed for some types of variables and distributions in the health inequality literature (e.g., see Erreygers 2009b, for the case of bivariate distributions in concentration analysis), to the best of our knowledge they have not been characterized in relation to explicit sets of axioms for individual bounded variables. Hence, after defining the concepts of *bipolar* and *almost‐bipolar* distributions, we show that the set of distributions reflecting maximum inequality for a given mean, depends on the axioms selected to rank these distributions of bounded cardinal variables. We show that, if we consider anonymity and the transfers principle alone, then the MIDs for a given mean are either *bipolar* or *almost‐bipolar* (Proposition 2). We show in Section 5 that, if we add the population principle to anonymity and the transfers principle, then for a given mean the subset of MIDs narrows down to just bipolar distributions.

We introduce some additional necessary notation. For some n∈N\{1}, let us denote a set of n−1 equally‐spaced grid points by

$$G_{n}=\left\{\frac{\left(n-1\right)L+U}{n},\frac{\left(n-2\right)L+2U}{n},\text{…},\frac{L+\left(n-1\right)U}{n}\right\}.$$

For example, for L=0, U=1 and n=4, $G_{4}=\left\{0.25,0.5,0.75\right\}$.

***Bipolar distribution:*** A distribution $x\in X_{n;D}$ is *bipolar* whenever for some $n^{\prime }\in N$ such that $n^{\prime }<n$, $n^{\prime }$ units in x attain the value of U and the remaining $n-n^{\prime }$ units attain the value of L.

Bipolar distributions consist of units with values at either the lower bound or upper bound exclusively, with at least one unit at each bound. Since $n^{\prime }$ could take any value between 1 and n−1, for any bipolar distribution $x\in X_{n;D}$, μ(x) is an element in $G_{n}$. For example, with L=0 and U=1, consider distribution x=(0.1,0.4,0.7,0.8), where μ(x)=0.5 is an element in $G_{4}$. An MID for x can be obtained by a sequence of regressive transfers until (or unless) no further regressive transfers are possible. Thus, the set of MIDs for x contains all possible permutations of the distribution $\hat{x}=\left(0,0,1,1\right)$. All distributions in the set are bipolar since two elements are equal to the lower bound of 0 and two elements are equal to the upper bound of 1.

***Almost‐bipolar distribution:*** A distribution $x\in X_{n;D}$ is *almost‐bipolar* whenever for some $n^{\prime }\in N\cup \left\{0\right\}$ such that $n^{\prime }<n$, $n^{\prime }$ units in x attain the value of U, $n-n^{\prime }-1$ units in x attain the value of L, and the leftover unit's attained value, which is necessarily $\varepsilon =n\mu \left(x\right)-n^{\prime }U-(n-n^{\prime }-1)L$, lies between L and U.

Almost bipolar distributions consist of all units with either the lower or upper bound value, except for one unit with an interior value of ε∈(L,U). If $y\in X_{n;D}$ is an almost‐bipolar distribution, then μ(y) cannot be an element of $G_{n}$. For instance, consider distribution y=(0.2,0.4,0.7,0.9), where μ(y)=0.55 with L=0 and U=1, which is not an element in $G_{4}$. Again, an MID for y can be obtained by a sequence of regressive transfers until (or unless) no further regressive transfers are possible. The corresponding MIDs, in this case, are all possible permutations of the distribution $\hat{y}=\left(0,0.2,1,1\right)$, with the leftover element being ε=0.2. Notably, $\hat{y}$ is almost‐bipolar.

Formally, we denote the set of all possible almost‐bipolar distributions by A⊂X; the set of all possible bipolar distributions by B⊂X; and the set of all distributions that are either bipolar or almost bipolar by M=A∪B. We use subscripts n, D and • and superscript μ(x) with A, B and M as we have done for X in order to define relevant subsets. Finally, we define, for every $x\in X_{n;D}$, a partially ordered set $\left(X_{n;D}^{\mu \left(x\right)}{,⪰}_{n}\right)$ such that for any pair $y,z\in X_{n;D}^{\mu \left(x\right)}$: (1) $z{≻}_{n}y$, which reads “z is more unequal than y,” if z is obtained from y through a sequence of regressive transfers with or without additional permutations and (2) $z{∼}_{n}y$, which reads “z is as unequal as y” if z is obtained from y only through a sequence of permutations.

Based exclusively on anonymity and the transfers principle, Proposition 2 establishes the existence of a set of *maximum‐inequality distributions* (MIDs) and shows that the set of MIDs associated to any distribution $x\in X_{n;D}$ contains permutations of either a bipolar or an almost‐bipolar distribution with population size n and same mean μ(x), that is, $M_{n;D}^{\mu \left(x\right)}=X_{n;D}^{\mu \left(x\right)}\cap M$.

Proposition 2 For any n∈N\{1}, any D∈D and for any $x\in X_{n;D}$ such that $\mu \left(x\right)\in \left(L,U\right)\cap Q_{++}$, a set of maximum inequality distributions $M_{n,D}^{\mu \left(x\right)}=X_{n;D}^{\mu \left(x\right)}\cap M$ constituting the maximal elements of the partially ordered set $\left(X_{n;D}^{\mu \left(x\right)}{,⪰}_{n}\right)$ exists and the elements of $M_{n;D}^{\mu \left(x\right)}$ are *bipolar* when $\mu \left(x\right)\in G_{n}$ or *almost‐bipolar* when $\mu \left(x\right)\notin G_{n}$.

See Appendix A2.

It is worth noting that the elements included in $M_{n;D}^{\mu \left(x\right)}$ are unique up to permutations; that is, given any two elements $x,y\in M_{n;D}^{\mu \left(x\right)}$, then y=xP for some permutation matrix P. Even though MIDs are hypothetical distributions unlikely to be observed in practice, they do represent the benchmark case of maximum inequality against which we can compare distributions of bounded variables sharing the same mean. The latter's inequality evaluations cannot be larger than their MID's as long as an inequality index I satisfies anonymity and the transfer principle.

### 3.1 Comparing MIDs With Different Means

Recall from the cake‐cutting illustration for non‐bounded variables in the introduction that the ranking of the most unequal distributions (i.e., one person owning everything), with different means, depends on the selected approach to inequality measurement. For instance, such MIDs are judged equally unequal by the relative Lorenz ordering and by most Lorenz‐consistent relative inequality measures, but absolute inequality indices and partial orderings deem MIDs with higher means more unequal. By contrast and as elucidated, the concept of maximum inequality changes in the presence of an upper bound. How should we compare the MIDs of bounded variables for different means given that they all reflect maximum inequality? We draw ethical intuitions from a parallel literature assessing inequality changes for non‐bounded variables due to a sequence of population shifts between a better‐off group and a worse‐off group owing to social progress (Temkin 1986) or, analogously, between a low‐income sector and a high‐income sector owing to income growth (Fields 1987, 1993, 1998; Amiel and Cowell 1994; Bosmans 2007).

Suppose there are two groups in a society: one better off (e.g., high‐income) and one worse off (e.g., low‐income). Moreover, let everyone within each group be equally well off, so that there is no inequality within each of the two. Suppose further that there are n people in the society and consider the following n−1 situations. In the first (i.e., the *initial*) situation, there are n−1 persons in the worse‐off group and only one person in the better‐off group; in the second situation, one person moves from the worse‐off group to the better‐off group, and so there are n−2 persons in the worse‐off group and two persons in the better‐off group; and so on. Finally, in the ${\left(n-1\right)}^{\text{th}}$ (i.e., the *final*) situation, there is only one person in the worse‐off group and the other n−1 persons are in the better‐off group. As we gradually move from the initial situation to the final situation, the mean gradually improves, but how should inequality change?

Five possible ethical judgments have been discussed in the literature as the mean improves along with the shift of population from the worse‐off group to the better‐off group:

1. An increase in inequality throughout;
2. A decrease in inequality throughout;
3. An initial increase in inequality, then a reduction after a certain point where inequality is maximized (i.e., an inverted U‐shape);
4. An initial reduction in inequality, then an increase after a certain point where inequality is minimized (i.e., a U‐shape);
5. No change in inequality.

Temkin (1986) and Fields (1998) both argue on the possibility for inequality to be increasing throughout (as an ever smaller number of people become victimized through the *isolation of the poor*) as well as the possibility for inequality to decrease throughout (reflecting a diminished *elitism of the rich* and the steady decrease in the number of those worse off). Temkin (1986) and Fields (1998), however, disagree on the possibilities of a U‐shape or an inverted‐U‐shape relationship. Temkin (1986) argues in favor of the possibility of an inverted‐U‐shape relationship, whereby the “isolation of the poor” judgment is claimed to dominate the “elitism of the rich” judgment when the mean is low, but the “elitism of the rich” judgment dominates at high mean levels. Meanwhile, Fields (1998) argues the exact opposite to justify a U‐shape relationship. Nevertheless, Bosmans (2007) shows that quasi‐concave inequality measures (comprising numerous relative, absolute and intermediate inequality measures) allow only the first three possibilities, that is, increasing throughout, decreasing throughout and the inverted‐U shape.

Thus, the literature features various arguments around the first four ethical judgments, in the case of non‐bounded variables, wherein none of the n−1 situations discussed above corresponds to maximum inequality (unless the two incomes are assumed unique). Importantly, a key difference emerges here for bounded variables, as for the latter each of the n−1 situations corresponds to a bipolar MID (i.e., the most unequal distribution) for a particular mean. Take the hypothetical case of 100 people (i.e., n=100), who may experience only one of the two extreme health alternatives (i.e., bounds): best health and worst health.^17^ Although this yields 99 bipolar MIDs, consider the following two distributions: A with 10 people experiencing best health and 90 people experiencing worst health; and B with 20 people experiencing best health and 80 people experiencing worst health. How should we compare a move from A to B? The “isolation of the poor” ethical judgment may suggest a rise in inequality as B reflects a situation with fewer people with worst health, but the “elitism of the rich” ethical judgment may suggest a reduction in inequality as 10 people previously experiencing worst health now experience best health.

A broader related question is how inequality ought to change when the mean gradually grows from the initial MID (i.e., one person with best health and 99 with worst health) to the final MID (99 people with best health and one with worst health). The most frequently used absolute inequality measures increase in value as the mean raises from the initial MID, reach their maximum values around the middle value of the mean (i.e., 50 people with best health and 50 people with worst health), and then decrease as the mean keeps growing towards the final MID; thereby depicting a Kuznets curve. Assessments based on most absolute inequality measures, thus, follow the third ethical judgment. Whichever the choice of inequality measure in the literature, we have not found compelling ethical justifications for ranking two MIDs differently despite their key common definitional trait; namely that in both distributions inequality cannot increase any further through regressive transfers (hence, we cannot even obtain one MID from another through such transfers). As is well known, we cannot transform one egalitarian distribution into another egalitarian distribution with a different mean through progressive transfers; yet we rank them equally. Hence, by analogy, we may justify ranking MIDs with different means in the same way.

As Temkin (1986, 118) eloquently stated, “two judges who accepted bribes in all of their cases might be equally corrupt, even if one tried fewer cases.” Temkin presented this argument while arguing in favor of the fifth ethical judgment. Thus, following Temkin (1986) and the aforementioned reasons, we propose considering the MIDs as equally unequal irrespective of their proportions of people with values in the lower and upper bounds. Since existing inequality indices do not satisfy the fifth ethical judgment (Bosmans 2007), we operationalize the latter with a property called the *maximality principle* as follows.

***Maximality principle:*** For any $x,y\in X_{n;D}$, I(x)=I(y) whenever $x\in M_{n;D}^{\mu \left(x\right)}$ and $y\in M_{n;D}^{\mu \left(y\right)}$.

The property requires that, whenever we pick any two (non‐trivial) MIDs, the corresponding levels of inequality must coincide. Stated otherwise, whenever no further regressive transfers can be performed, then we have reached maximal inequality *irrespective of the mean of the distribution*.

## 4 The Class of Normalized Indices for Comparisons With Fixed Population Size

Building on the key properties introduced in Section 2 and the maximality principle in 3, we characterize a new class of inequality indices. We show that, within our framework, inequality should be measured as an increasing function of observed inequality as a proportion of the maximum inequality level reachable given a mean attainment. Theorem 1 presents our proposed family of inequality indices for comparisons with fixed population sizes, which we call the class of *normalized inequality indices*.

Theorem 1 For any n∈N\{1} and any $x\in X_{n;•}$, an inequality index I satisfies anonymity, the transfer principle, the equality principle, the maximality principle, strong consistency and cardinal invariance if and only if there exist a positive finite constant M, a strictly Schur‐convex function $f:X_{n;•}\to R_{+}$, and an increasing function $H:R\to R_{+}$ such that:

(1) $$I\left(x\right)=H\left(\left[H^{-1}\left(M\right)-H^{-1}\left(0\right)\right]\frac{f\left(x\right)-f\left(\bar{x}\right)}{f\left(\hat{x}\right)-f\left(\bar{x}\right)}+H^{-1}\left(0\right)\right),$$

where $\bar{x}=\mu \left(x\right)1_{n}$ is the egalitarian distribution with the same mean as x, and $\hat{x}\in M_{n;•}^{\mu \left(x\right)}$ is an MID for x. Additionally, f satisfies the following two restrictions:

(2) $$\frac{f\left(x^{S}\right)-f\left({\bar{x}}^{S}\right)}{f\left({\hat{x}}^{S}\right)-f\left({\bar{x}}^{S}\right)}=\frac{f\left(x\right)-f\left(\bar{x}\right)}{f\left(\hat{x}\right)-f\left(\bar{x}\right)},$$

and, for some constants $\lambda \in Q_{++}$ and $\delta \in Q_{+}$:

(3) $$\frac{f\left(\lambda x+\delta \right)-f\left(\lambda \bar{x}+\delta \right)}{f\left(\lambda \hat{x}+\delta \right)-f\left(\lambda \bar{x}+\delta \right)}=\frac{f\left(x\right)-f\left(\bar{x}\right)}{f\left(\hat{x}\right)-f\left(\bar{x}\right)}.$$

See Appendix A3.

According to Theorem 1, a normalized inequality index I(x) in our proposed class evaluated at distribution x is an increasing function (i.e., H) of any symmetric and S‐convex function f(x) evaluated at x, *subtracted* by its corresponding minimum possible value $f\left(\bar{x}\right)$ evaluated at the egalitarian distribution $\bar{x}$, and then normalized by the *difference* between its corresponding maximum possible value $f\left(\hat{x}\right)$ evaluated at any of its uniquely associated MIDs, namely $\hat{x}\in M_{n;D}^{\mu \left(x\right)}$, and its corresponding minimum possible value $f\left(\bar{x}\right)$.

For all practical purposes, we advocate using H(z)=z and M=1 in Equation (1), which yields a functionally simpler subclass of inequality indices from the class characterized in Theorem 1. Indeed, with such choices, the indices in our proposed class take the form:

(4) $$I\left(x\right)=\frac{f\left(x\right)-f\left(\bar{x}\right)}{f\left(\hat{x}\right)-f\left(\bar{x}\right)}.$$

Moreover, when the chosen f is an admissible traditional inequality index fulfilling the equality principle (such as the Gini coefficient or the coefficient of variation), then $f\left(\bar{x}\right)=0$ and the ratio on the right‐hand side of Equation (4) further simplifies to:

(5) $$I\left(x\right)=\frac{f\left(x\right)}{f\left(\hat{x}\right)}.$$

Conveniently, the values of the inequality measures in Equations (4) and (5) range between *zero* in the absence of inequality (i.e., $x=\bar{x}$) and *one* for an MID (i.e., $x=\hat{x}$). In fact, the indices in subclass (5) measure inequality as a proportion of the maximum attainable with f for a given mean. More generally, every normalized inequality index in the characterized class shares some key features, starting with increasing in value after a regressive transfer and decreasing owing to a progressive transfer.

Secondly, the value of every normalized index remains unchanged across attainment and shortfall representations (i.e., satisfying strong consistency) because the condition in Equation (2) is satisfied. More precisely in terms of the subclasses in Equations (4) and (5), the condition in Equation (2) states that I(x) is strongly consistent if and only if the ratios on the right‐hand sides satisfy perfect complementarity. Thus notably, f does not need to satisfy perfect complementarity itself in order to secure the strong consistency of a normalized inequality index.

Thirdly, the value of every normalized index remains the same for two distributions when one is obtained from the other through a positive linear transformation of the measurement unit (i.e., satisfying cardinal invariance) because the condition in Equation (3) is satisfied. As with strong consistency, the condition in Equation (3) states that I is cardinally invariant if and only if the ratios on the right‐hand sides of Equations (4) and (5) satisfy cardinal invariance. Again notably, f itself does not need to be cardinally invariant in order to guarantee the cardinal invariance of a normalized inequality index.

Crucially, f admits numerous functional forms. For instance, f can take the form of absolute inequality indices in the rank‐dependent class characterized by Lambert and Zheng (2011, theorem 4) as the following two conditions hold for all its members (such as the absolute Gini index): $f\left(x^{S}\right)=f\left(x\right)$ and f(λx+δ)=λf(x). These two conditions clearly comply with the restrictions in Equations (2) and (3). Additionally, f can also adopt the forms of the absolute inequality indices in the rank‐independent class characterized by Lambert and Zheng (2011, theorem 4) if they are based on homogeneous functions (as with the variance and the standard deviation, for instance). Indeed, the following two conditions hold for homogeneous members of the rank‐independent class characterized by Lambert and Zheng (2011): $f\left(x^{S}\right)=f\left(x\right)$ and f(λx+δ)=g(λ)f(x), where $g:R_{++}\to R_{++}$. Clearly, these two conditions also comply with the restrictions in Equations (2) and (3).

Every relative inequality index expressible as the product of an homogeneous Lambert‐Zheng absolute inequality index times a function of the mean, is also admissible. For instance, when f is either the Gini coefficient (which is equal to the absolute Gini index divided by the mean) or the coefficient of variation (which is equal to the standard deviation divided by the mean), we obtain $f\left(x^{S}\right)=\frac{\mu \left(x\right)}{U+L-\mu \left(x\right)}f\left(x\right)$ and $f\left(\lambda x+\delta \right)=\frac{\lambda \mu \left(x\right)}{\lambda \mu \left(x\right)+\delta }f\left(x\right)$, which again, satisfy Equations (2) and (3). The same results hold for every member of the Donaldson‐Weymark class of generalized Gini indices (Donaldson and Weymark 1980), confirming their admissibility. Similarly, all measures of the Lasso de la Vega and Aristondo (2012) class based on the aforementioned absolute and relative inequality indices are also admissible.

By contrast, no members of the rank‐independent Atkinson class (Atkinson 1970) are suitable for obtaining strongly consistent normalized inequality indices. Finally, only one member of the generalized entropy class (Shorrocks 1980) is an admissible functional form for f, namely the squared coefficient of variation.^18^

By way of examples, we present two normalized inequality indices, which consider two popular inequality measures as admissible functional forms for f, assuming H(z)=z and M=1 as in Equation (4), and $\mu \left(x\right)\in G_{n}$ (meaning that only bipolar MIDs are considered).^19^ Also for convenience of presentation, we refer to the normalized inequality index corresponding to the admissible form f as $f^{*}$ (instead of I), in order to clarify that $f^{*}$ is derived from an admissible f.

When f is the absolute or the relative Gini index, that is, $f\left(x\right)=G_{a}\left(x\right)$ or $f\left(x\right)=G_{r}\left(x\right)$, then it is easy to check (see Appendix A6) that

(6) $$G^{*}\left(x\right)=G_{a}^{*}\left(x\right)=G_{r}^{*}\left(x\right)=\frac{G_{a}\left(x\right)\left(U-L\right)}{\left(U-\mu \left(x\right)\right)\left(\mu \left(x\right)-L\right)}.$$

Thus, $G_{a}^{*}\left(x\right)$ compares $G_{a}\left(x\right)$ against the maximal inequality value that such index could possibly take for any distribution with mean equal to μ(x) (which equals $\frac{\left(U-\mu \left(x\right)\right)\left.\left(\mu \left(x\right)-L\right)\right)}{U-L}$ when the MID is bipolar, i.e., when $\mu \left(x\right)\in G_{n}$). Expectably, the normalized inequality indices derived from the absolute and the relative Gini indices coincide because the latter is a product of the former times a function depending on the mean, which cancels out. To simplify notation, such normalized Gini index will be referred to as $G^{*}\left(x\right)$.

We can also derive the normalized versions of the standard deviation $\left(f\left(x\right)=\sigma \left(x\right)=\sqrt{V\left(x\right)}\right)$ and the coefficient of variation (f(x)=CV(x)=σ(x)/μ(x)). It is easy to check (see Appendix A6) that $\sigma^{*}\left(x\right)=CV^{*}\left(x\right)$, that is,

(7) $$\sigma^{*}\left(x\right)=\frac{\sigma \left(x\right)}{\sqrt{\left(U-\mu \left(x\right)\right)\left(\mu \left(x\right)-L\right)}}.$$

Once again, the normalized version of an absolute inequality index and its relative counterpart coincide for the same aforementioned reason.

## 5 The Class of Normalized Indices for Comparisons With Varying Population Sizes

At least since Dalton (1920), the most popular answer to the challenge of comparing inequality across distributions with different population sizes is the *population principle*, which requires that identical cloning of all units should leave inequality unaltered (thereby rendering populations with different sizes comparable).^20^ A normalized inequality measure from the class in Theorem 1 does not comply with the population principle even when an admissible functional form of f does, because even though the replication of a bipolar MID is itself an MID, the replication of an almost bipolar MID is *not* an MID, based on how Proposition 2 defines an MID.^21^ Therefore, if we want our normalized inequality measures to fulfill the population principle we must adopt a different definition of the set of MIDs, one compliant with the population principle. Proposition 3 establishes the existence of such a set of MIDs, and shows that the set of MIDs, associated with all distributions *sharing the same mean* across *all population sizes* taking values within (L,U) is, in this case, equal to $B_{•;D}^{\mu \left(x\right)}=X_{•;D}^{\mu \left(x\right)}\cap B$.^22^ Based on the transfer and population principles combined with anonymity, these MIDs are defined as the distributions that maximize inequality among all possible distributions with the *same mean* but *varying population sizes*.

Likewise, we can order distributions across all population sizes. That is, for every $x\in X_{•;D}$, we can define a partially ordered set $\left(X_{•;D}^{\mu \left(x\right)},⪰\right)$ such that for any pair $y,z\in X_{•;D}^{\mu \left(x\right)}$: (1) z≻y, which reads “z is more unequal than y,” if z is obtained from y through a sequence of regressive transfers with or without additional permutations and/or replications and (2) z∼y, which reads “z is as unequal as y” if z is obtained from y only through a sequence of permutations and/or replications.

Proposition 3 For any D∈D and any $x\in X_{•;D}$ such that $\mu \left(x\right)\in \left(L,U\right)\cap Q_{++}$, a set of maximum inequality distributions $B_{•;D}^{\mu \left(x\right)}=X_{•;D}^{\mu \left(x\right)}\cap B$ constituting the maximal elements of the partially ordered set $\left(X_{•;D}^{\mu \left(x\right)},⪰\right)$ exists and all elements of $B_{•;D}^{\mu \left(x\right)}$ are *bipolar*.

See Appendix A4.

According to Proposition 3, in a setting compliant with the population principle, *only* bipolar distributions maximize inequality. Thus, the Maximality Principle introduced in Section 3 must be adapted and rewritten as follows:

***Restricted Maximality Principle:*** For any $x,y\in X_{•;D}$, I(x)=I(y) whenever $x\in B_{•;D}^{\mu \left(x\right)}$ and $y\in B_{•;D}^{\mu \left(y\right)}$.

Again, this principle states that whenever no further regressive transfers are feasible and we have reached a bipolar distribution, then inequality is maximal (no matter what the mean of the distribution is). With this reformulated version of the maximality principle, we can now axiomatically characterize the class of normalized inequality indices compliant with the population principle:

Theorem 2 For any x∈X, an inequality index I satisfies anonymity, the transfer principle, the equality principle, the *restricted* maximality principle, the *population principle*, strong consistency and cardinal invariance if and only if there exist a positive finite constant M, a strictly Schur‐convex function $f:X\to R_{+}$, and an increasing function $H:R\to R_{+}$ such that:

$$I\left(x\right)=H\left[\left(H^{-1}\left(M\right)-H^{-1}\left(0\right)\right)\frac{f\left(x\right)-f\left(\bar{x}\right)}{f\left(\hat{x}\right)-f\left(\bar{x}\right)}+H^{-1}\left(0\right)\right],$$

where $\bar{x}$ is the egalitarian distribution with the same mean as x, and $\hat{x}\in B^{\mu \left(x\right)}$ is a *bipolar* MID for x. Besides, f satisfies the population principle and the following two restrictions:

$$\frac{f\left(x^{S}\right)-f\left({\bar{x}}^{S}\right)}{f\left({\hat{x}}^{S}\right)-f\left({\bar{x}}^{S}\right)}=\frac{f\left(x\right)-f\left(\bar{x}\right)}{f\left(\hat{x}\right)-f\left(\bar{x}\right)},$$

and for any constants $\lambda \in Q_{++}$ and $\delta \in Q_{+}$:

$$\frac{f\left(\lambda x+\delta \right)-f\left(\lambda \bar{x}+\delta \right)}{f\left(\lambda \hat{x}+\delta \right)-f\left(\lambda \bar{x}+\delta \right)}=\frac{f\left(x\right)-f\left(\bar{x}\right)}{f\left(\hat{x}\right)-f\left(\bar{x}\right)}.$$

See Appendix A5.

Theorem 2 implies that the normalized inequality indices abide by the population principle (in addition to all the properties in Theorem 1) as long as f satisfies the population principle and is *evaluated at any bipolar distribution with mean equal to* μ(x). Good examples include the normalized inequality indices in Equations (6) and (7), already introduced in Section 4. The indices in Equations (6) and (7) can rank distributions with any mean whose value is different from either bound because both the absolute Gini index and the standard deviation (as well as the Gini index and the coefficient of variation) satisfy the population principle.

## 6 Further Comparative Insights

We now provide some insights into how the two proposed classes of normalized inequality indices (in Sections 4 and 5) compare with each other as well as how they both compare with standard absolute and relative measures. First, note that the two approaches to measuring normalized inequality (corresponding to the two definitions of MIDs and their respective classes of indices) bear a large degree of overlap. In fact, the formulas for normalized inequality indices compliant with the population principle (Section 5) is identical to the corresponding formulas for indices suitable for fixed population sizes (Section 4) whenever $\mu \left(x\right)\in G_{n}$.^23^ In fact, when the population size n is sufficiently large and the decimal precision is kept fixed (as is the case in many empirical applications), the condition $\mu \left(x\right)\in G_{n}$ is always satisfied.

**FIGURE 1:**
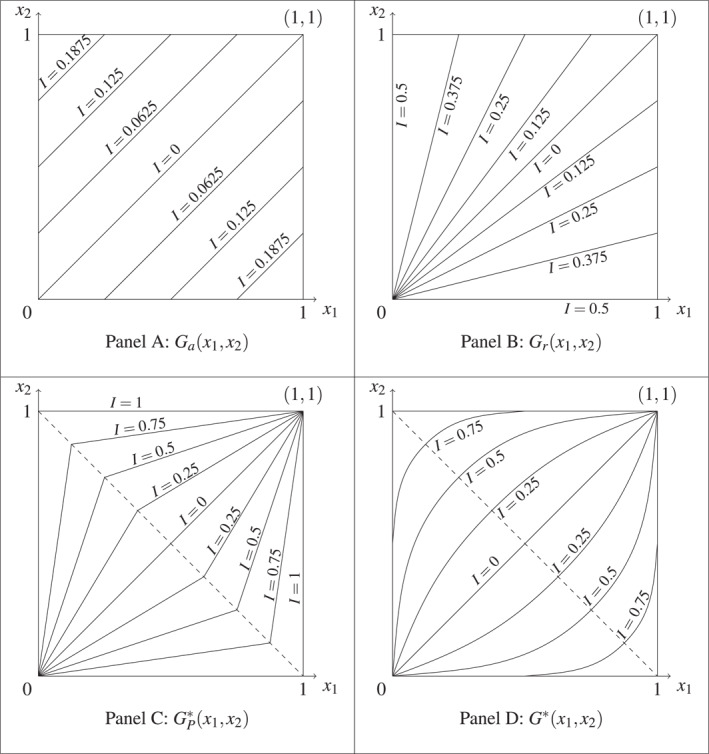
Iso‐inequality contours for the different Gini coefficient (n=2, L=0, and U=1). The figure is based on n=2 and U=1. $G_{a}\left(x_{1},x_{2}\right)$ in panel (A) is the absolute Gini index applied to an attainment distribution. $G_{r}\left(x_{1},x_{2}\right)$ in panel (B) is the relative Gini index applied to the same attainment distribution. $G_{P}^{*}\left(x_{1},x_{2}\right)$ in panel (C) is the normalized Gini index applied to the attainment distribution for fixed population. $G^{*}\left(x_{1},x_{2}\right)$ in panel (D) is the normalized Gini index applied to the attainment distribution for variable population.

In the context of n=2, we provide insights on how the different normalized inequality measures behave and compare vis‐a‐vis each other, and with respect to standard absolute and relative inequality measures using the Gini coefficient. The non‐trivial case with n=2 lays the foundation for how the corresponding inequality indices behave for the more general case of n>2. Furthermore, the simplicity of the n=2 setting allows a neat inspection of the iso‐inequality level contours, which can be thought as the fingerprint of the corresponding inequality measures.^24^ Figure 1 presents the iso‐inequality contours of the absolute Gini index ($G_{a}$, Figure 1 panel A), the relative Gini index ($G_{r}$, Figure 1 panel B), the normalized Gini index based on Theorem 1 (i.e., for fixed population; $G_{P}^{*}$ in Equation (A14) in Appendix A6, Figure 1 panel C), and the normalized Gini index complying with the population principle (the same formulation as $G^{*}$ in Equation (6), Figure 1 panel D), in the case where L=0 and U=1 (Appendix A7 shows how we arrive at these iso‐inequality contours).^25^

As is well‐known, $G_{a}\left(x_{1},x_{2}\right)\in \left[0,0.25\right]$ and the iso‐inequality contours for $G_{a}$ are parallel to the 45° line, while $G_{r}\left(x_{1},x_{2}\right)\in \left[0,0.5\right]$ and the iso‐inequality contours for $G_{r}$ are straight lines “emanating from” (or “converging to”) the origin (0, 0). In contrast, the iso‐inequality contours for the two normalized Gini indices, $G_{P}^{*}\left(x_{1},x_{2}\right)\in \left[0,1\right]$ and $G^{*}\left(x_{1},x_{2}\right)\in \left[0,1\right]$, exhibit completely different shapes. In the case of $G_{P}^{*}$, all level contours are made of two line segments meeting in the diagonal $\left\{\left(x_{1},x_{2}\right)\in {\left[0,1\right]}^{2}\;|\;x_{1}+x_{2}=1\right\}$, which, together, connect the points (0, 0) and (1, 1). Their shapes (though not their corresponding inequality levels) coincide with the level contours of $G_{r}\left(x_{1},x_{2}\right)$ when $\mu \left(x_{1},x_{2}\right)\leq 1/2$ and with those of $G_{r}\left(x_{1}^{S},x_{2}^{S}\right)$ when $\mu \left(x_{1},x_{2}\right)\geq 1/2$ (where $x_{1}^{S}=1-x_{1}$ and $x_{2}^{S}=1-x_{2}$, see Appendix A7). In addition, one has that $G_{P}^{*}\left(x_{1},x_{2}\right)=G_{P}^{*}\left(x_{1}^{S},x_{2}^{S}\right)$. Lastly, the level contours $G^{*}\left(x_{1},x_{2}\right)=c$ (where c∈[0,1]) are curves that (i) are symmetrical with respect to the $x_{2}=1-x_{1}$ axis for all c∈[0,1] (i.e., $G^{*}\left(x_{1},x_{2}\right)=G^{*}\left(x_{1}^{S},x_{2}^{S}\right)$) and (ii) they connect the points (0, 0) and (1, 1) when c≤1/2.

As can be inferred from Figure 1 panel C, all the distributions $\left(x_{1},x_{2}\right)$ lying at the border of the unit square maximize inequality (i.e., they are MIDs) when the latter is measured with $G_{P}^{*}\left(x_{1},x_{2}\right)$. By contrast, Figure 1 panel D shows that, when the population principle is imposed, only the bipolar distributions, namely (0, 1) and (1, 0), maximize inequality. The relative Gini index shown in panel B $\left(G_{r}\right)$ is the only measure in Figure 1 that fails to be strongly consistent. As expected from Proposition 1, the absolute $\left(G_{a}\right)$ and normalized Gini indices ($G_{P}^{*}$, $G^{*}$) not only satisfy the strong consistency axiom, but also its more stringent version, perfect complementarity. This happens for all values of n≥2 and for the two normalized inequality measures explored in this paper: the normalized Gini index and the normalized standard deviation (see equations (6) and (7)). The variegated shapes of the iso‐inequality contours when moving from one inequality measure to another (see Figure 1) explain the discrepancies that might exist among them.

## 7 Empirical Illustration: Cross‐Country Inequality Trends in Three Health Indicators

In order to illustrate the empirical relevance of our proposal, we study the evolution of cross‐country inequality in three health indicators relevant to the United Nation's 3rd Sustainable Development Goal (SDG), “Ensure healthy lives and promote well‐being for all at all ages”: the under‐5 survival rates (indicator 3.2.1; target 3.2; SDG 3), the infant survival rates (related to target 3.2; SDG 3) and the Hepatitis B (HepB3) immunization coverage rates among 1‐year‐olds (related to target 3.3, SDG 3).^26^ We select the under‐five survival rate and the infant survival rate, namely the attainment complements of the respective mortality rates. The data come from the United Nations' Department of Economic and Social Welfare website.^27^ We obtain the survival rates by subtracting the mortality rates from 1000 and then normalizing the differences by 1000. Therefore, the survival rates also lie between zero and one. The data are available for 201 countries.

**FIGURE 2:**
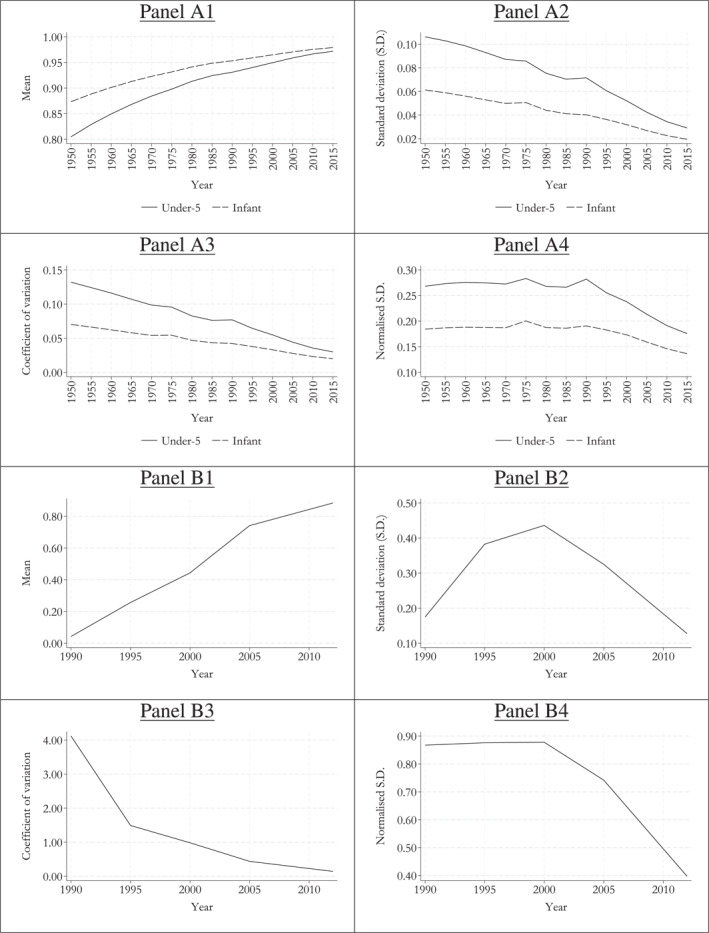
Change in cross‐country mean and standard deviation, coefficient of variation and normalized standard deviation for the health indicators. The graphs in panels (A1–A4) are based on the data from 201 countries; whereas, the graphs in panels (B1–B4) are based on the data from 152 countries. *Source:* Authors' own computations.

Panel A1 in Figure 2 shows the change in mean attainments for the two selected child survival indicators between 1950 and 2015, for every 5‐year period.^28^ All global averages display steady improvements since 1950. The mean under‐5 survival rate increases from 0.80 in 1950 to 0.97 in 2015, whereas the mean infant survival rate increases from 0.87 in 1950 to 0.98 in 2015. That is, both means lie well above 0.50 throughout the studied period.

Figure 2 panel A2 presents the trends in standard deviation since 1950 for the two child survival indicators. Predictably for standard deviation when the growth in the mean is far from egalitarian, the values decrease throughout the period as mean attainment, already well above 0.5, gets closer to its upper bound. Figure 2 panel A3 shows the evolution of relative inequality measured by the coefficient of variation. Again, predictably for this index if the growth in the mean is far from egalitarian, relative inequality decreases throughout.^29^

Finally, Figure 2 panel A4 presents the trends in normalized standard deviation, that is, $\sigma^{*}$ in Equation (7). Unlike both standard deviation (absolute) and coefficient of variation (relative), the normalized standard deviation registers a non‐upward trend for both health indicators through the first decades of the studied period. Indeed, for both indicators, the normalized standard deviation remains stable until nearly 1990, and falls thereafter. That is, in both cases, we conclude that for most of the 20th century, cross‐country inequality in the two child survival indicators remains stable compared to its maximum possible value (which was decreasing, according to the standard deviation and coefficient of variation), and then its level falls gradually in the 21st century (as maximum absolute and relative values kept decreasing with increasing means). Thus, our empirical illustration shows that normalized indices can produce different inequality trends vis‐a‐vis their traditional absolute and relative counterparts.

By way of another corroborating example, panel B1 in Figure 2 shows the change in mean attainments for HepB3 infant immunization rates. Unlike the two previous indicators, here mean attainment is observed increasing from 0.04 in 1990 (i.e., very close to the lower bound) to 0.88 in 2012 (not far from the upper bound either). Correspondingly, Figure 2 panel B2 displays a Kuznets curve for HepB3 immunization rates using standard deviation, while Figure 2 panel B3 shows a steady decline in the coefficient of variation (relative). Unless the growth in mean immunization rates remains egalitarian throughout the periods, these two inequality trends are largely predictable since the maximum values of standard deviation and the coefficient of variation describe Kuznets curves and downward sloping lines, respectively.^30^ But then, interestingly, Figure 2 panel B4 shows that the normalized standard deviation remains stable initially and then slopes downward throughout.

Here, the main lesson from the normalized standard deviation is that the increase in absolute inequality in immunization rates experienced during the 20th century was similar in comparison to the increase in maximum absolute inequality, hence the stability in normalized inequality. However, normalized inequality declined unabated into the 21st century. Meanwhile, we also learn from the normalized standard deviation that the observed decline in the coefficient of variation (relative) was similar in comparison to the decline in its maximum value as mean attainment grew in the 20th century; but then the coefficient of variation declined faster in comparison to its maximum possible values. In conclusion, the normalized index shows that the diffusion of HepB3 vaccination became more egalitarian as the attained proportion of maximum possible inequality decreased with the years in the 21st century (and with further increases in mean immunization rates).

## 8 Concluding Remarks

Bounded variables are fundamentally different from unbounded variables as the former cannot increase or decrease infinitely. Consequently, whenever the mean of a distribution moves closer to any of its bounds, the level of inequality assessed by several traditional inequality measures may fall simply because there is not enough room for variation. The concept of maximum feasible inequality with bounded variables is also quite different from maximum feasible inequality in the context of unbounded variables.^31^ We propose a new approach to assessing inequality for individual bounded variables relying on a new property called the *maximality principle*, which demands that the distributions of bounded variables reflecting maximum feasible inequality be ranked equally. We propose two new classes of inequality indices. The maximality principle leads to a type of normalization, whereby each inequality measure in our proposed classes is an increasing function of observed inequality levels compared against the maximum inequality level achievable with the same measure across all hypothetical distributions having the same mean. Furthermore, our proposed classes of normalized inequality indices evaluate inequality across attainment and shortfall representations consistently.

To illustrate the empirical relevance of our methodological proposal, we examine the evolution of cross‐country inequality in three health indicators, comparing the normalized standard deviation against its absolute and relative counterparts (i.e., standard deviation and coefficient of variation, respectively). The normalized standard deviation portrays a markedly different picture of cross‐country evolution of inequality compared to the patterns produced by the absolute and relative counterparts. More specifically, in the case of the two child survival indicators, both the standard deviation and the coefficient of variation decrease as mean attainment approaches its upper bound, but the trends based on normalized standard deviation suggest that global progress did not follow more conceivably egalitarian paths until the 1990s. By contrast, in the case of the hepatitis‐B immunization rate, for mean attainment > 50% the normalized standard deviation shows that the decline in observed inequality measured by the absolute and relative counterparts was greater than the contemporaneous decline in the corresponding maximum inequality values; thus uncovering inequality improvement above and beyond what would be expected from the predictable narrowing in maximum possible dispersion as mean attainment tends toward its upper bound.

Future research could consider the normative foundations and feasibility of classes of indices for bounded variables based on the relaxation of strong consistency in favor of inconsistent or weakly consistent inequality measurement (i.e., following Bosmans 2016), combined alternatingly with different ethical rules for ranking MIDs, including inter alia Erreygers (2009b), Bosmans (2007) and our maximality principle. Likewise, future research could explore partial orderings respecting the properties that were combined to generate the normalized inequality indices. Furthermore, there remain other measurement challenges in the context of bounded variables. For example, Lasso de la Vega and Aristondo (2012) provide conditions whose fulfillment guarantees robustness of inequality comparisons to changes in the upper bound. Though admittedly this problem is not that serious when bounds are neither arbitrary nor expected to change across time and space (e.g., in the case of indicators expressed as percentage ratios), it is nonetheless worth exploring how our proposed measurement framework could accommodate such potential concern.

## Conflicts of Interest

The authors declare no conflicts of interest.

## Data Availability

The data that support the findings of this study are available from the corresponding author upon reasonable request.

## Endnotes

^1^
Besides indicators of health, examples of non‐pecuniary variables include indicators of education, political freedom, democracy level, freedom from violence, happiness and life satisfaction, trust, corruption, household or environmental characteristics, access to services, poverty, socio‐demographic characteristics, etc.

^2^
We acknowledge the existence of potential alternative value judgments for the ranking of MIDs (Erreygers 2009b; Kjellsson and Gerdtham 2013), which in turn may lead to different axiomatic characterizations.

^3^
We denote sets of real, rational and natural numbers by R, Q and N, respectively. The non‐negative and strictly positive counterparts are represented by adding the ${subscripts}_{+}$ ${and}_{++}$, respectively.

^4^
The density of rational numbers within the set of real numbers makes the rationality of L and U inconsequential for practical applications.

^5^
When the mean is equal to the lower or upper bound, namely μ(x)=L or μ(x)=U (i.e., the excluded cases), one normative option is to rank them equally vis‐a‐vis other egalitarian distributions with L<μ(x)<U. Whichever the normative choice, we do not rank these distributions with extreme means using our proposed class of continuous inequality indices.

^6^
Formally, for some $x,y\in X_{n;D}$, y is obtained from x by a *progressive transfer* whenever there are two units i,j and some $k\in Q_{++}$ such that $y_{i}=x_{i}+k\leq x_{j}-k=y_{j}$ and $y_{l}=x_{l}$ for every l≠i,j. Alternatively, y is obtained from x by a *regressive transfer* whenever there are two units i,j and some $k\in Q_{++}$ such that $y_{i}+k=x_{i}\leq x_{j}=y_{j}-k$ and $y_{l}=x_{l}$ for every l≠i,j.

^7^
A *permutation matrix* is a square matrix with exactly one element in each row and column equal to 1, and the rest of the elements equal to zero.

^8^
Some of the bounded indicators discussed in this paper are not literally transferable. For instance, we do not consider worsening the HepB3 vaccination rates in high‐vaccination countries and transferring vaccinated children to countries with worse vaccination coverage. Yet, one can compare two hypothetical scenarios, for example pre‐ and post‐“progressive transfers,” and still judge the latter exhibiting lower inequality than the former.

^9^
For some $x,y\in X_{n;D}$, y is majorised by x if $\sum_{i=1}^{j}y_{\left[i\right]}\leq \sum_{i=1}^{j}x_{\left[i\right]}$ for all j=1,…,n−1 and $\sum_{i=1}^{n}y_{i}=\sum_{i=1}^{n}x_{i}$, where $x_{\left[i\right]}$ and $y_{\left[i\right]}$ are the $i^{\text{th}}$ largest elements of x and y, respectively (Dahl 2001, 113).

^10^
By contrast, strictly convex functions do not satisfy the transfer principle unless they are also symmetric, in which case they are strictly Schur‐convex. Meanwhile, strictly quasi‐convex functions do not satisfy the transfer principle unless they are also convex and symmetric, in which case, again, they are strictly Schur‐convex.

^11^
For any $\lambda \in Q_{++}$, $\lambda 1_{n}$ is the constant or egalitarian distribution where all n elements are equal to λ.

^12^
Lambert and Zheng (2011) and Bosmans (2016) provide good examples of the spectrum of approaches, among others. Zheng (2007) and Lambert and Zheng (2011) propose weaker requirements for the consistency of inequality rankings across different measurement units.

^13^
Arguably, the concern for different degrees of consistency may be more pressing when both representations (attainment and shortfall) can be deemed “different sides of the same coin” (Clarke et al. 2002, 1927), warranting equal attention.

^14^
We note that Bosmans (2016) introduced a less frequently invoked consistency property, known as *weak consistency*, which is predicated on the possibility of finding pairs of “different” inequality indices for consistent comparisons as long as one index is used for the attainment distribution and a separate index is used for the shortfall counterpart.

^15^
Lambert and Zheng (2011, theorem 4) showed that two broad classes of absolute inequality indices satisfy strong consistency if and only if they comply with perfect complementarity. Hence, Proposition 1 is more general as it applies to all inequality indices.

^16^
Distribution $y\in X_{n^{\prime };D}$ for some $n^{\prime }=\gamma n$ and γ∈N\{1} is said to be obtained from $x\in X_{n;D}$ by a *population replication*, whenever y=(x,…,x), that is, γ copies of x are repeated one after the other in y.

^17^
This example is inspired by Erreygers (2009b, 508).

^18^
Proofs available upon request.

^19^
Examples featuring $\mu \left(x\right)\notin G_{n}$ appear in Appendix A6.

^20^
For a more general proposal, see Aboudi et al. (2010). The population principle is defined in Section 2.

^21^
For instance, when n=2, L=0 and U=1, an MID associated to a distribution with mean equal to 0.25 is (0, 0.5). However, the replication (0, 0.5, 0, 0.5) of that MID is not an MID itself. The corresponding MID for a distribution with n=4 and with mean equal to 0.25 is in fact (0, 0, 0, 1).

^22^
Recall that B is the set of all bipolar distributions.

^23^
Readers are reminded of our examples of normalized inequality indices in Section 4, whose formulas vary depending on whether $\mu \left(x\right)\notin G_{n}$ (i.e., almost bipolar MIDs) or $\mu \left(x\right)\in G_{n}$ (i.e., bipolar MIDs).

^24^
Indeed, the behavior of some very popular inequality measures like the Gini index or the Variance (which can be defined on a recursive basis) is entirely determined by what happens in the most basic case n=2 (see details in Ebert 2010).

^25^
Results remain essentially unaltered when the absolute and relative Gini indices are substituted by the standard deviation and the coefficient of variation, respectively.

^26^

https://sdgs.un.org/goals/goal3.

^27^
Source of the mortality data is https://population.un.org/wpp/Download/Standard/Mortality/ and the Hepatitis B (HepB3) immunization coverage rates among 1‐year‐olds is https://data.un.org/Data.aspx?q=Hepatitis+B&d=WHO&f=MEASURE_CODE%3aWHS4_117.

^28^
The mean attainment does not include population weights. Each country, irrespective of its size, is considered as a unit with equal importance.

^29^
The maximum values of the (relative) coefficient of variation decrease monotonically as the mean grows from the lower to the upper bound of the variable.

^30^
Derivation and figures available upon request.

^31^
In the unbounded setting (i.e., in the context of income inequality), Milanovic et al. (2011) suggest and discuss the notion of “Inequality Possibility Frontier” (IPF), which measures the maximum level of inequality that is potentially attainable for a given level of average income. In that setting, such IPF is reached whenever a vast majority of lower‐class individuals survive at subsistence levels and a small elite accumulates the reminder of total income.

## Appendix A1 Proof of Proposition 1

The “if” part is straightforward. For the “only if” part consider the definition of strong consistency: $I\left(x\right)\leq I\left(y\right)\Leftrightarrow I\left(x^{S}\right)\leq I\left(y^{S}\right)$ for any $x,y\in X_{n;D}$. Now let $y=x^{S}$. Then we get: $I\left(x\right)\leq I\left(x^{S}\right)\Leftrightarrow I\left(x^{S}\right)\leq I\left(x\right)$ which can only hold if $I\left(x\right)=I\left(x^{S}\right)$.

## Appendix A2 Proof of Proposition 2

Let us start with an $x\in X_{n;D}\backslash M$ (i.e., x is neither bipolar nor almost bipolar) for some n∈N\{1} such that $\mu \left(x\right)\in \left(L,U\right)\cap Q_{++}$. Given that in the proposition's partial order $\left(X_{n;D}^{\mu \left(x\right)}{,⪰}_{n}\right)$, a regressive transfer increases inequality while a permutation keeps it unaltered and both keep the mean unaltered, we may always perform a sequence of regressive transfers (with or without additional permutations) until exhaustion to obtain any element of M that belongs in the set of distributions with the same population size and the same mean, namely $X_{n;D}^{\mu \left(x\right)}$.^32^

Now, there can be two cases: (i) $\mu \left(x\right)\in G_{n}$ and (ii) $\mu \left(x\right)\notin G_{n}$, where

$$G_{n}=\left\{\frac{\left(n-1\right)L+U}{n},\text{…},\frac{L+\left(n-1\right)U}{n}\right\}$$

is the set of n−1 equally‐spaced grid points between L and U.

**Case (i)**: Whenever $\mu \left(x\right)\in G_{n}$, then there exists a natural number $n^{\prime }\leq n$ such that $\mu \left(x\right)=[(n-n^{\prime })L+n^{\prime }U]/n$. Starting with x, it is possible to have a series of regressive transfers until a distribution with $n^{\prime }$ elements equaling U and $n-n^{\prime }$ elements equaling L is reached. In this case, the set of MIDs is $X_{n;D}^{\mu \left(x\right)}\cap B$.

**Case (ii)**: Whenever $\mu \left(x\right)\notin G_{n}$, then there exist a natural number $n^{\prime }\leq n$ such that $[(n-n^{\prime })L+n^{\prime }U]/n<\mu \left(x\right)<[(n-n^{\prime }-1)L+(n^{\prime }+1)U]/n$. In this case, a series of regressive transfers are possible until $n^{\prime }$ elements are equal to U and $n-n^{\prime }-1$ elements are equal to L. Note that it is not possible for $n^{\prime }+1$ elements to be equal to U because $\mu \left(x\right)<[(n-n^{\prime }-1)L+(n^{\prime }+1)U]/n$. However, when $n^{\prime }$ elements are equal to U, then $\mu \left(x\right)>[(n-n^{\prime }-1)L+n^{\prime }U]/n$. Therefore, the remaining element will have a value of $\varepsilon =n\mu \left(x\right)-n^{\prime }U-(n-n^{\prime }-1)$ so that: $\mu \left(x\right)=n^{\prime }\times U/n+\left(n-n^{\prime }-1\right)\times L/n+\varepsilon /n$. It is straightforward to verify that ε∈(L,U). In this case, the set of MIDs is $X_{n,D}^{\mu \left(x\right)}\cap A$.

Thus, the maximum inequality distribution (MID) for x is an element in the set $X_{n;D}^{\mu \left(x\right)}\cap \left(A\cup B\right)=X_{n;D}^{\mu \left(x\right)}\cap M$, which by our definition is equal to $M_{n;D}^{\mu \left(x\right)}$. Now, whenever $x\in X_{n;D}\cap M$ for some n∈N\{1} (i.e., x is either bipolar or almost bipolar), it can be trivially checked that $x\in M_{n;D}^{\mu \left(x\right)}$. Hence, a set of MIDs for any $x\in X_{n;D}$ such that μ(x)∈(L,U) always exists and constitutes the set of maximal elements $M_{n;D}^{\mu \left(x\right)}$ of the partially ordered set $\left(X_{n;D}^{\mu \left(x\right)}{,⪰}_{n}\right)$.

## Appendix A3 Proof of Theorem 1

We first prove the *sufficiency* part. Consider some $x\in X_{n;D}$ for some n∈N\{1} and some D∈D. So, the set of corresponding MIDs is $M_{n;D}^{\mu \left(x\right)}$ by Proposition 2. We then already know that

(A1)
$$I\left(x\right)=H\left[\left(H^{-1}\left(M\right)-H^{-1}\left(0\right)\right)\frac{f\left(x\right)-f\left(\bar{x}\right)}{f\left(\hat{x}\right)-f\left(\bar{x}\right)}+H^{-1}\left(0\right)\right]\;\text{if}\;\;x\in X_{n;D}$$

where $\bar{x}=\mu \left(x\right)1_{n}$, $\hat{x}\in M_{n;D}^{\mu \left(x\right)}$, $f:X_{n;D}\to R_{+}$ is a strictly Schur‐convex function satisfying the functional restriction in Equations (2) and (3).

We now show that I satisfies the required properties. (i) Consider any $x\in X_{n;D}$. Since $f\left(\hat{x}\right)-f\left(\bar{x}\right)>0$ because any $\hat{x}\in M_{n;D}^{\mu \left(x\right)}$ can be obtained from $\bar{x}$ by a series of regressive transfers (i.e., $\bar{x}$ is majorized by $\hat{x}$) and f is strictly Schur convex, it follows directly from the formulation in Equation (A1) that I satisfies the *equality principle* as $I\left(\bar{x}\right)=0$ (because $\left[f\left(\bar{x}\right)-f\left(\bar{x}\right)\right]/\left[f\left(\hat{x}\right)-f\left(\bar{x}\right)\right]=0$), and the *maximality principle* as $I\left(\hat{x}\right)=I\left(\hat{y}\right)=M$ for any $\hat{x}\in M_{n;D}^{\mu \left(x\right)},\hat{y}\in M_{n;D}^{\mu \left(y\right)}$ (because $\left[f\left(\hat{x}\right)-f\left(\bar{x}\right)\right]/\left[f\left(\hat{x}\right)-f\left(\bar{x}\right)\right]=1$).

(ii) Suppose $y\in X_{n;D}$ is obtained from x such that y=xP, where P is a permutation matrix. By definition, μ(x)=μ(y), $M_{n;D}^{\mu \left(y\right)}=M_{n;D}^{\mu \left(x\right)}$ and $\bar{y}=\bar{x}$. Since f is symmetric by virtue of being strictly Schur convex (Roberts and Varberg 1973), f(y)=f(x) and also $f\left(\hat{y}\right)=f\left(\hat{x}\right)$ for any $\hat{x},\hat{y}\in M_{n;D}^{\mu \left(x\right)}$. So, I(y)=I(x). Thus I satisfies *anonymity*.

(iii) Suppose $y^{\prime }\in X_{n;D}$ is obtained from x by a regressive transfer (i.e., x is majorized by $y^{\prime }$). Again, by definition, $\mu \left(x\right)=\mu (y^{\prime })$, $M_{n;D}^{\mu \left(y^{\prime }\right)}=M_{n;D}^{\mu \left(x\right)}$ and ${\bar{y}}^{\prime }=\bar{x}$. Provided f is strictly Schur‐convex, $f(y^{\prime })>f\left(x\right)$ and so $I(y^{\prime })>I\left(x\right)$. Thus, I satisfies the *transfer principle*.

(iv) Suppose $y\in X_{n;D}$ is obtained from x in such a way that $y=x^{S}$. If f satisfies Equation (2), after basic simplification we get $I\left(x^{S}\right)=I\left(x\right)$, which according to Proposition 1, is equivalent to *strong consistency*.

(v) Suppose $y\in X_{n;•}$ is obtained from x in such a way that $y=\lambda x+\delta 1_{n}$ with λ>0 and δ≥0. If f satisfies Equation (3), after basic simplification we get I(y)=I(x). Then I(λx+δ)=I(x) for any rational constants λ>0 and δ≥0 implies cardinal invariance.

Observe that in this part of the proof, we start with a fixed $D=\left[L,U\right]\cap Q_{+}$, but compliance with cardinal invariance extends the result to all D∈D.

Let us now prove the *Necessity* part. Suppose I satisfies anonymity. Then, by definition of anonymity, I must be symmetric. Now, in order to show that I must be strictly Schur‐convex, let us assume the contrary. That is, imagine I is not strictly Schur‐convex. In that case, by definition, there is some $x,y\in X_{n;D}$ such that x majorizes y but I(x)≤I(y). Additionally, we know that any sequence of progressive transfers (from x to y) induces a majorisation of the post‐transfers distribution (y) by the pre‐transfers distribution (x) (Marshall and Olkin 1979, 6) and vice versa. Thus, I violates the transfer principle. So, if I is symmetric and satisfies the transfer principle, then it must be strictly Schur‐convex.

Then, given that a monotonically increasing transformation of a strictly Schur‐convex function is also strictly Schur‐convex, we may write (without loss of generality) I(x)=H[af(x)+b] for some $x\in X_{n;D}$, where $a\in R_{++}$, b∈R, f is some strictly Schur‐convex function, and H:R→R is a strictly increasing function.

Next, the equality principle requires that $I\left(\bar{x}\right)=H\left[af\left(\bar{x}\right)+b\right]=0$, therefore $af\left(\bar{x}\right)+b=H^{-1}\left(0\right)$ or

(A2)
$$b=H^{-1}\left(0\right)-af\left(\bar{x}\right).$$

We now proceed in four steps to prove that I(x) is a bounded function.

**Step 1**: To simplify our analysis, we extend the domain of our inequality function I to the set of real numbers. Given the continuity of I and the density of the set of rational numbers into the real numbers, it is possible to *univocally* extend the domain of I (which is $X_{n;D}$) into ${\bar{X}}_{n;D}$ (i.e., the closure of $X_{n;D}$, which corresponds to ${\left[L,U\right]}^{n}$).^33^ That is, it is possible to define a continuous function (denoted as $\bar{I}$) that univocally extends I to the set of real numbers. By construction, one has that $\bar{I}{|}_{X_{n;D}}=I$ (i.e., whenever $\bar{I}$ is restricted to rational numbers, then it coincides with I).

**Step 2**: Let $x\in {\bar{X}}_{n;D}$. We prove that ${\bar{X}}_{n;D}^{\mu \left(x\right)}$ is a bounded and closed set within ${\bar{X}}_{n;D}$. To do so, we need to define a topology and a distance function in ${\bar{X}}_{n;D}$.^34^ We can simply use the subspace topology of $R^{n}$ for ${\bar{X}}_{n;D}$. Thus, if $B_{r}\left(x\right)$ is the standard open n−ball in $R^{n}$ with center x and radius r>0, then we define the open n−balls in ${\bar{X}}_{n;D}$ as ${\tilde{B}}_{r}\left(x\right)≔B_{r}\left(x\right)\cap {\bar{X}}_{n;D}$. As per distance function, for any $x,y\in {\bar{X}}_{n;D}$ we can use $d\left(x,y\right)=\sqrt{\sum_{i}{\left(x_{i}-y_{i}\right)}^{2}/n}$, which is a variant of the standard Euclidean distance that normalizes by the number of observations (n), and complies with the standard distance function axioms (in footnote 34).

Using d(⋅,⋅) as a distance function, the maximal possible distance between two points within ${\bar{X}}_{n;D}$ equals U−L (i.e., it is the distance that obtains between any two distributions $a,b\in {\bar{X}}_{n;D}$ such that $a_{i},b_{i}\in \left\{L,U\right\}$ and $a_{i}+b_{i}=U+L$ for all i∈{1,…,n}). Thus, the distance between any other two points in ${\bar{X}}_{n;D}$ must be smaller than U−L. Hence, the open ball ${\tilde{B}}_{U-L}\left(x\right)$ contains the entire set ${\bar{X}}_{n;D}$ (i.e., ${\tilde{B}}_{U-L}\left(x\right)⊃{\bar{X}}_{n;D}$), so ${\bar{X}}_{n;D}$ is a bounded set. Since ${\bar{X}}_{n;D}^{\mu \left(x\right)}\subset {\bar{X}}_{n;D}$, ${\bar{X}}_{n;D}^{\mu \left(x\right)}$ is also bounded.

We now prove that ${\bar{X}}_{n;D}^{\mu \left(x\right)}$ is closed. To do that, we prove that ${\bar{X}}_{n;D}\backslash {\bar{X}}_{n;D}^{\mu \left(x\right)}$ is open. Let $y\in {\bar{X}}_{n;D}\backslash {\bar{X}}_{n;D}^{\mu \left(x\right)}$ (i.e., μ(y)≠μ(x)), and define $\varepsilon ≔\left|\mu \left(y\right)-\mu \left(x\right)\right|/2$. Then, $\left.{\tilde{B}}_{\varepsilon }\left(y\right)\right)\subset {\bar{X}}_{n;D}\backslash {\bar{X}}_{n;D}^{\mu \left(x\right)}$, so we have defined an open n−ball centered in y that is completely included within ${\bar{X}}_{n;D}\backslash {\bar{X}}_{n;D}^{\mu \left(x\right)}$. Thus, ${\bar{X}}_{n;D}\backslash {\bar{X}}_{n;D}^{\mu \left(x\right)}$ is open, so ${\bar{X}}_{n;D}^{\mu \left(x\right)}$ is closed.

Hence, we have proved that ${\bar{X}}_{n;D}^{\mu \left(x\right)}$ is a **compact** set within ${\bar{X}}_{n;D}$.

**Step 3**: Since $\bar{I}:{\bar{X}}_{n;D}\to R_{+}$ is a continuous function, and ${\bar{X}}_{n;D}^{\mu \left(x\right)}$ is a compact set, then $\bar{I}\left({\bar{X}}_{n;D}^{\mu \left(x\right)}\right)$ (i.e., the image of ${\bar{X}}_{n;D}^{\mu \left(x\right)}$ by $\bar{I}$) is a compact set. Compact sets within $R_{+}$ are bounded, which implies there exist $m_{x},M_{x}\in R_{+}$ such that $m_{x}\leq \bar{I}\left(x\right)\leq M_{x}$ for all $x\in {\bar{X}}_{n;D}^{\mu \left(x\right)}$.

**Step 4**: Imposing the equality principle implies $m_{x}=0$ for all $x\in {\bar{X}}_{n;D}$. In addition, $M_{x}=\bar{I}\left(\hat{x}\right)$ for all $x\in {\bar{X}}_{n;D}$. Applying the maximality principle, it turns out that $M_{x}=\bar{I}\left(\hat{x}\right)=\bar{I}\left(\hat{y}\right)=M_{y}$ for all $x,y\in {\bar{X}}_{n;D}$. Now, define $M≔M_{x}$. Thus, for all $x\in {\bar{X}}_{n;D},0\leq \bar{I}\left(x\right)\leq M$. Finally, since $\bar{I}$ is an extension of I, one also has that 0≤I(x)≤M for all $x\in X_{n;D}$ (i.e., we have proven that I(x) is a bounded function).

Now, since $I\left(\hat{x}\right)=M$ and $b=H^{-1}\left(0\right)-af\left(\bar{x}\right)$ (from Equation A2), we have that $af\left(\hat{x}\right)+b=af\left(\hat{x}\right)-af\left(\bar{x}\right)+H^{-1}\left(0\right)=H^{-1}\left(M\right)$. Thus, $a=\left(H^{-1}\left(M\right)-H^{-1}\left(0\right)\right)/\left(f\left(\hat{x}\right)-f\left(\bar{x}\right)\right)$, so

(A3)
$$I\left(x\right)=H\left[\left(H^{-1}\left(M\right)-H^{-1}\left(0\right)\right)\frac{f\left(x\right)-f\left(\bar{x}\right)}{f\left(\hat{x}\right)-f\left(\bar{x}\right)}+H^{-1}\left(0\right)\right]$$

whenever $x\in X_{n;D}$.

Then, from Proposition 1, we conclude that if I is strongly consistent, then $I\left(x^{S}\right)=I\left(x\right)$, which in turn means:

$$\frac{f\left(x^{S}\right)-f\left({\bar{x}}^{S}\right)}{f\left({\hat{x}}^{S}\right)-f\left({\bar{x}}^{S}\right)}=\frac{f\left(x\right)-f\left(\bar{x}\right)}{f\left(\hat{x}\right)-f\left(\bar{x}\right)}.$$

For the last part of the proof, we note from Equation (A3) that satisfaction of cardinal invariance implies:

$$\frac{f\left(\lambda x+\delta \right)-f\left(\lambda \bar{x}+\delta \right)}{f\left(\lambda \hat{x}+\delta \right)-f\left(\lambda \bar{x}+\delta \right)}=\frac{f\left(x\right)-f\left(\bar{x}\right)}{f\left(\hat{x}\right)-f\left(\bar{x}\right)}.$$

Like in the sufficiency part of the proof, in the necessity part we also start with a fixed domain $D=\left[L,U\right]\cap Q_{+}$, but compliance with cardinal invariance extends the result to all D∈D.

## Appendix A4 Proof of Proposition 3

Consider an $x\in X_{n;D}\subset X$ for some n∈N\{1} and some D∈D such that $\mu \left(x\right)\in \left(L,U\right)\cap Q_{++}$; recalling that $L\in Q_{+}$, $U\in Q_{++}$ and 0≤L<U. Based on Proposition 2, there can be two possible cases: (i) $\mu \left(x\right)\in G_{n}$, or (ii) $\mu \left(x\right)\notin G_{n}$.

**Case (i)**: if $\mu \left(x\right)\in G_{n}$, then we know from Proposition 2 that the MID for x (i.e., $\hat{x}$) exists, and is bipolar.

**Case (ii)**: if $\mu \left(x\right)\notin G_{n}$, then we know from Proposition 2 that the MID for x (i.e., $\hat{x}$) exists, and is almost‐bipolar. Then $\hat{x}$ has $n^{\prime }$ number of U’s, $n-n^{\prime }-1$ number of L’s and a ε such that $\left.L<\varepsilon <U\right)$ and $\varepsilon \in Q_{++}$. We will now show that through a sufficient number of population replications and regressive transfers, it is always possible to obtain some y from x such that the corresponding $\hat{y}$ is bipolar and yet μ(y)=μ(x).

In $\hat{x}$, given that both $\varepsilon ,U\in Q_{++}$, it is clearly the case that $\varepsilon /U\in Q_{++}$. Consequently, there always exist some γ,p∈N such that ε/U=p/γ, or equivalently γε=pU. Given that ε<U, it is always true that γ>1. Suppose, y is obtained from x by replicating xγ times. Recalling that $\varepsilon =n\mu \left(x\right)-n^{\prime }U-(n-n^{\prime }-1)L$, we obtain after replication the following sum across γ individuals with value ε in y: $\gamma \varepsilon =n\gamma \mu \left(x\right)-n^{\prime }\gamma U-(n-n^{\prime }-1)\gamma L=pU$. Since the mean has not changed, we can solve from that sum for μ(y)=μ(x) in order to obtain:

$$\mu \left(y\right)=\frac{\left(n^{\prime }\gamma +p\right)U+\left(n-n^{\prime }-1\right)\gamma L}{n\gamma }.$$

That is, unlike $\hat{x}$, the corresponding $\hat{y}$ must only consist of $(n-n^{\prime }-1)\gamma$ number of L’s and $\left(n^{\prime }\gamma +p\right)$ number of U’s. Hence, $\hat{y}$ is bipolar.

In both cases, we have proved that, when imposing the population principle, the MIDs for x exist and consist of bipolar distributions.

## Appendix A5 Proof of Theorem 2

We first prove the sufficiency part. Applying Theorem 1, which holds for $X_{n;D}$, we can show that I satisfies *anonymity*, the *transfer principle* and the *equality principle*. Now, since $f\left(\hat{x}\right)>f\left(\bar{x}\right)$ for any $\hat{x}\in B_{•;D}^{\mu \left(x\right)}$ because any $\hat{x}\in B_{•;D}^{\mu \left(x\right)}$ can be obtained from $\bar{x}$ by a series of regressive transfers (i.e., $\bar{x}$ is majorized by $\hat{x}$) with or without combinations of replications and permutations, and f is strictly Schur‐convex, we have $I\left(\hat{x}\right)=M$; that is, I satisfies the *restricted maximality principle*. Finally, we prove that I satisfies the population principle. Let y be obtained from $x\in X_{n;D}$ through a replication. Then, by definition, μ(x)=μ(y) and so by Proposition 3, $B_{•;D}^{\mu \left(x\right)}=B_{•;D}^{\mu \left(y\right)}$. It is also straightforward to verify that $\bar{y}$ is a replication of $\bar{x}$. Therefore, based on $\left(X_{•;D}^{\mu \left(x\right)},⪰\right)$, y∼x and $\bar{y}∼\bar{x}$, and hence $f\left(\bar{y}\right)=f\left(\bar{x}\right)$ and f(y)=f(x) because f satisfies the population principle. Coupled with $f\left(\hat{y}\right)=f\left(\hat{x}\right)$ for any $\hat{x},\hat{y}\in B_{•;D}^{\mu \left(x\right)}$ and $f\left(\hat{x}\right)-f\left(\bar{x}\right)>0$, clearly I(y)=I(x). Hence, I satisfies the population principle. Finally, satisfaction of strong consistency and cardinal invariance follows the reasoning of points (iv) and (v) in the proof of Theorem 1.

The proof of the necessity part is similar to the proof of Theorem 1's necessity part, with some modifications. If I satisfies anonymity and the transfer principle, then, without loss of generality, I(x)=H[af(x)+b] for $x\in X_{n;D}$, where $a\in R_{++}$, b∈R, f is some Strictly Schur‐convex function and H is a strictly increasing function. Suppose that $y\in X_{n^{\prime };D}$ is obtained from x by *replication* for some $n^{\prime }=\alpha n$, where α∈N\{1}. Given that I satisfies the population principle, then I(y)=I(x). It follows that f(y)=f(x) since a>0 and so f also satisfies the population principle. By the equality principle, which requires that $I\left(\bar{x}\right)=0$ where $\bar{x}=\mu \left(x\right)1_{n}$, we obtain $af\left(\bar{x}\right)+b=H^{-1}\left(0\right)$ or $b=H^{-1}\left(0\right)-af\left(\bar{x}\right)$.

Mimicking the proof of Theorem 1, we now show that I(x) is a bounded function. Recall that we denote by $\bar{I}$ the unique extension of I to the set of real numbers. Since ${\bar{X}}_{n;D}^{\mu \left(x\right)}$ is compact for all n∈N\{1} (see Theorem 1) and ${\bar{X}}_{•;D}^{\mu \left(x\right)}=\cup_{n}{\bar{X}}_{n;D}^{\mu \left(x\right)}$ is a countable union of compact sets, then ${\bar{X}}_{•;D}^{\mu \left(x\right)}$ is also compact. Since the extension to the set of real numbers of I (i.e., $\bar{I}$) is a continuous function, then $\bar{I}\left({\bar{X}}_{•;D}^{\mu \left(x\right)}\right)$ is bounded. Thus, there exist $m_{x},M_{x}\in R_{+}$ such that $m_{x}\leq \bar{I}\left(x\right)\leq M_{x}$ for all $x\in {\bar{X}}_{•;D}^{\mu \left(x\right)}$. By the Equality Principle, $m_{x}=0$ for all $x\in {\bar{X}}_{•;D}$. By the Restricted Maximality Principle, $M_{x}=\bar{I}\left(\hat{x}\right)=\bar{I}\left(\hat{y}\right)=M_{y}$ for all $x,y\in {\bar{X}}_{•;D}$. Now, define $M≔M_{x}$. Thus, for all $x\in {\bar{X}}_{•;D},0\leq \bar{I}\left(x\right)\leq M$, so $\bar{I}$ is bounded, and I must be bounded as well. Now, since $I\left(\hat{x}\right)=M$ and $b=H^{-1}\left(0\right)-af\left(\bar{x}\right)$, we have that $af\left(\hat{x}\right)+b=af\left(\hat{x}\right)-af\left(\bar{x}\right)+H^{-1}\left(0\right)=H^{-1}\left(M\right)$. Thus, $a=\left(H^{-1}\left(M\right)-H^{-1}\left(0\right)/\left(f\left(\hat{x}\right)-f\left(\bar{x}\right)\right)\right.$, so we obtained the desired functional form whenever $x\in X_{•;D}$.

Like in Theorem 1, here we also start the proof with a fixed domain $D=\left[L,U\right]\cap Q_{+}$, but compliance with cardinal invariance extends the result to all D∈D. Equations (2) and (3) are demonstrated with the respective parts of the proof for Theorem 1, completing the proof.

## Appendix A6 Derivation of Normalized Inequality Indices

### The Case of Bipolar MIDs

Here we show the derivation of the inequality measures presented as examples in Section 4. We start with the formulas relying on bipolar MIDs followed by formulas based on almost bipolar MIDs. The former are simpler than the latter.

In a bipolar MID $\hat{x}$, assume that a share s of the population attains the value of L and the rest (1−s) the value of U. Given that $\mu \left(\hat{x}\right)=\mu \left(x\right)$, by definition, the following restriction must hold:

(A4)
$$\mu \left(\hat{x}\right)=s\times L+\left(1-s\right)\times U\Rightarrow s=\frac{U-\mu \left(x\right)}{U-L}.$$

#### The Absolute Gini Index

Computing the absolute Gini index for $\hat{x}$ yields:

(A5)
$$G_{a}\left(\hat{x}\right)=s\left(1-s\right)\left(U-L\right).$$

Plugging Equation (A4) into Equation (A5) and manipulating algebraically yields:

$$G_{a}\left(\hat{x}\right)=\frac{U-\mu \left(x\right)}{U-L}\left(1-\frac{U-\mu \left(x\right)}{U-L}\right)\left(U-L\right)=\frac{\left(U-\mu \left(x\right)\right)\left(\mu \left(x\right)-L\right)}{U-L}.$$

#### The Relative Gini Index

Computing the relative Gini index for $\hat{x}$ yields:

(A6)
$$G_{r}\left(\hat{x}\right)=\frac{s\left(1-s\right)\left(U-L\right)}{\mu \left(x\right)}.$$

Plugging Equation (A4) into Equation (A6) and manipulating algebraically yields:

$$G_{r}\left(\hat{x}\right)=\frac{\left(\mu \left(x\right)-L\right)\left(U-\mu \left(x\right)\right)}{\mu \left(x\right)\left(U-L\right)}.$$

#### The Standard Deviation

Computing the standard deviation for $\hat{x}$ yields:

(A7)
$$\sigma \left(\hat{x}\right)=\sqrt{s{\left[\mu \left(x\right)-L\right]}^{2}+\left(1-s\right){\left[U-\mu \left(x\right)\right]}^{2}}.$$

Plugging Equation (A4) into Equation (A7) and manipulating algebraically, we obtain:

$$\sigma \left(\hat{x}\right)=\sqrt{\left[U-\frac{\mu \left(x\right)}{U-L}\right]{\left[\mu \left(x\right)-L\right]}^{2}+\frac{\mu \left(x\right)-L}{U-L}{\left[U-\mu \left(x\right)\right]}^{2}}=\sqrt{\left[\mu \left(x\right)-L\right]\left[U-\mu \left(x\right)\right]}.$$

#### The Coefficient of Variation

Computing the coefficient of variation for $\hat{x}$ yields:

(A8)
$$CV\left(\hat{x}\right)=\frac{\sigma \left(\hat{x}\right)}{\mu \left(\hat{x}\right)}=\frac{\sqrt{\left[\mu \left(x\right)-L\right]\left[U-\mu \left(x\right)\right]}}{\mu \left(x\right)}.$$

### The Case of Almost Bipolar MIDs

In an almost bipolar MID $\hat{x}$ we have $n^{\prime }$ units in the population with value U, one unit with value 0<ε<U and the rest, $n-n^{\prime }-1$ with value L. Moreover, $\varepsilon =n\mu \left(x\right)-n^{\prime }U-\left(n-n^{\prime }-1\right)L$. For each of the denominators of the indices mentioned in Section 4 we get:

#### The Absolute Gini Index

(A9)
$$G_{a}\left(\hat{x}\right)=\frac{1}{2n^{2}}\left[\left(n-n^{\prime }-1\right)\times 1\times |L-\varepsilon |+\left(n^{\prime }\right)\times 1\times |U-\varepsilon |+n^{\prime }\left(n-n^{\prime }-1\right)\times 1\times |L-U|\right]$$

Simplifying Equation (A9) we get the denominator of Equation (A14) for the almost bipolar case (noting later that the 2 in the fraction gets canceled out as it also appears in the numerator's formula):

(A10)
$$G_{a}\left(\hat{x}\right)=\frac{1}{2n^{2}}\left[\left(n-n^{\prime }-1\right)\left(\varepsilon -L\right)+\left(n^{\prime }\right)\left(U-\varepsilon \right)+n^{\prime }\left(n-n^{\prime }-1\right)\left(U-L\right)\right].$$

#### The Relative Gini Index

Essentially we get the same formula for the denominator of Equation (A14) as in Equation (A9), but divided by μ(x) (again, the 2 in the fraction gets canceled out as it also appears in the numerator's formula):

(A11)
$$G_{r}\left(\hat{x}\right)=\frac{1}{2n^{2}\mu \left(x\right)}\left[\left(n-n^{\prime }-1\right)\left(\varepsilon -L\right)+\left(n^{\prime }\right)\left(U-\varepsilon \right)+n^{\prime }\left(n-n^{\prime }-1\right)\left(U-L\right)\right].$$

#### The Standard Deviation

(A12)
$$\sigma \left(\hat{x}\right)=\sqrt{\frac{1}{n}\left[\left(n-n^{\prime }-1\right){\left(L-\mu \left(x\right)\right)}^{2}+n^{\prime }{\left(U-\mu \left(x\right)\right)}^{2}+{\left(\varepsilon -\mu \left(x\right)\right)}^{2}\right]}.$$

Simplifying Equation (A12) we get the denominator of Equation (A15) for the almost bipolar case.

#### The Coefficient of Variation

We get the same formula as in Equation (A12) but divided by μ(x):

(A13)
$$CV\left(\hat{x}\right)=\frac{1}{\mu \left(x\right)}\sqrt{\frac{1}{n}\left[\left(n-n^{\prime }-1\right){\left(L-\mu \left(x\right)\right)}^{2}+n^{\prime }{\left(U-\mu \left(x\right)\right)}^{2}+{\left(\varepsilon -\mu \left(x\right)\right)}^{2}\right]}.$$

Finally, for each of the aforementioned indices (for bipolar and almost bipolar MIDs), we compute $f\left(x\right)/f\left(\hat{x}\right)$.

### Examples for Comparisons With Fixed Population Sizes

#### The Absolute and Relative Gini Indices

For any $x\in X_{n;D}$, $G_{a}^{*}\left(x\right)=G_{r}^{*}\left(x\right)=G_{p}^{*}\left(x\right)$, where

(A14)
$$G_{P}^{*}\left(x\right)=\left\{\begin{array}{cc} \frac{G_{a}\left(x\right)\left(U-L\right)}{\left(\mu \left(x\right)-L\right)\left(U-\mu \left(x\right)\right)}\; & \text{if}\;\mu \left(x\right)\in G_{n}\\ \; & \\ \frac{G_{a}\left(x\right)n^{2}}{\left(n-n^{\prime }-1\right)\left(\varepsilon -L\right)+n^{\prime }\left(n-n^{\prime }-1\right)\left(U-L\right)+n^{\prime }\left(U-\varepsilon \right)}\; & \text{if}\;\mu \left(x\right)\notin G_{n}\\ \; & \end{array}\right..^{35}$$

#### The Standard Deviation and the Coefficient of Variation

For any $x\in X_{n;D}$,

(A15)
$$\sigma_{P}^{*}\left(x\right)=\left\{\begin{array}{cc} \frac{\sigma \left(x\right)}{\sqrt{\left(\mu \left(x\right)-L\right)\left(U-\mu \left(x\right)\right)}}\; & \text{if}\;\mu \left(x\right)\in G_{n}\\ \; & \\ \frac{\sigma \left(x\right)\sqrt{n}}{\sqrt{\left(n-n^{\prime }-1\right){\left(L-\mu \left(x\right)\right)}^{2}+n^{\prime }{\left(U-\mu \left(x\right)\right)}^{2}+{\left(\varepsilon -\mu \left(x\right)\right)}^{2}}}\; & \text{if}\;\mu \left(x\right)\notin G_{n}\\ \; & \end{array}\right..$$

## Appendix A7 Derivation of Iso‐Inequality Contours

When n=2, L=0 and U=1, the absolute Gini index can be written as $G_{a}\left(x_{1},x_{2}\right)=|x_{1}-x_{2}|/4$, where $\left(x_{1},x_{2}\right)\in {\left[0,1\right]}^{2}$. In that case, the iso‐inequality contours are straight lines parallel to the 45° line. In this setting, the relative Gini index can be written as $G_{r}\left(x_{1},x_{2}\right)=|x_{1}-x_{2}|/4\mu =|x_{1}-x_{2}|/2\left(x_{1}+x_{2}\right)$. Since this function is homogeneous of degree 0 (i.e., $G_{r}\left(\lambda x_{1},\lambda x_{2}\right)=G_{r}\left(x_{1},x_{2}\right)$ for all λ>0), the iso‐inequality contours are straight lines emanating from (or converging to) the origin (0, 0).

What about the iso‐inequality contours for the normalized Gini index that does not comply with the population principle $\left(G_{P}^{*}\left(x_{1},x_{2}\right)\right)$? Here, $G_{2}=\left\{1/2\right\}$, so there are basically two cases: either $\mu \left(x_{1},x_{2}\right)\leq 1/2$ or $\mu \left(x_{1},x_{2}\right)\geq 1/2$. Case (i): $\mu \left(x_{1},x_{2}\right)=\left(x_{1}+x_{2}\right)/2\leq 1/2$. Here, the MIDs associated with $\left(x_{1},x_{2}\right)$ are $\left\{\left(0,x_{1}+x_{2}\right),\left(x_{1}+x_{2},0\right)\right\}$. When the absolute Gini index is applied to any of those distributions, one obtains $G_{a}\left(0,x_{1}+x_{2}\right)=G_{a}\left(x_{1}+x_{2},0\right)=\left(x_{1}+x_{2}\right)/4$. Hence, $G_{P}^{*}\left(x_{1},x_{2}\right)=|x_{1}-x_{2}|/\left(x_{1}+x_{2}\right)$. These are straight lines emanating from (or converging to) the origin (0, 0). Case (ii): $\mu \left(x_{1},x_{2}\right)=\left(x_{1}+x_{2}\right)/2\geq 1/2$. Now, the MIDs associated with $\left(x_{1},x_{2}\right)$ are $\left\{\left(x_{1}+x_{2}-1,1\right),\left(1,x_{1}+x_{2}-1\right)\right\}$. Calculating the absolute Gini index of any of those distributions yields $G_{a}\left(x_{1}+x_{2}-1,1\right)=G_{a}\left(1,x_{1}+x_{2}-1\right)=\left(2-\left(x_{1}+x_{2}\right)\right)/4$. Hence $G_{P}^{*}\left(x_{1},x_{2}\right)=|x_{1}-x_{2}|/\left(2-\left(x_{1}+x_{2}\right)\right)=|x_{1}^{S}-x_{2}^{S}|/\left(x_{1}^{S}+x_{2}^{S}\right)$ (where $x_{1}^{S}=1-x_{1}$ and $x_{2}^{S}=1-x_{2}$). These are straight lines emanating from (or converging to) the point (1, 1). Finally, it is easy to prove that the two sets of iso‐inequality contours match at the intersection (i.e., for the set of points $\left\{\left(x_{1},x_{2}\right)\in {\left[0,1\right]}^{2}|x_{1}+x_{2}=1\right\}$, the values of the iso‐inequality contours examined in cases (i) and (ii) coincide).

According to Theorem 2 and Equation (6), the normalized Gini index complying with the population principle is simply defined as $G^{*}\left(x_{1},x_{2}\right)=G_{a}\left(x_{1},x_{2}\right)/\left(\mu \left(x_{1},x_{2}\right)\left(1-\mu \left(x_{1},x_{2}\right)\right)\right)$. Manipulating algebraically, one obtains that $G^{*}\left(x_{1},x_{2}\right)=|x_{1}-x_{2}|/\left(\left(x_{1}+x_{2}\right)\left(2-\left(x_{1}+x_{2}\right)\right)\right)$. This is the function from which the iso‐inequality contours shown in Figure 1 panel D are calculated.
